# Aspartate or arginine? Validated redox state X-ray structures elucidate mechanistic subtleties of Fe^IV^ = O formation in bacterial dye-decolorizing peroxidases

**DOI:** 10.1007/s00775-021-01896-2

**Published:** 2021-09-03

**Authors:** Marina Lučić, Michael T. Wilson, Dimitri A. Svistunenko, Robin L. Owen, Michael A. Hough, Jonathan A. R. Worrall

**Affiliations:** 1 School of Life Sciences, University of Essex, Wivenhoe Park, Colchester, CO4 3SQ UK; 2 Present Address: Diamond Light Source, Harwell Science and Innovation Campus, Didcot, OX11 0DE Oxfordshire UK

**Keywords:** Heme peroxidase, Ferryl, X-ray free electron laser, Kinetic isotope effect, Serial crystallography

## Abstract

Structure determination of proteins and enzymes by X-ray crystallography remains the most widely used approach to complement functional and mechanistic studies. Capturing the structures of intact redox states in metalloenzymes is critical for assigning the chemistry carried out by the metal in the catalytic cycle. Unfortunately, X-rays interact with protein crystals to generate solvated photoelectrons that can reduce redox active metals and hence change the coordination geometry and the coupled protein structure. Approaches to mitigate such site-specific radiation damage continue to be developed, but nevertheless application of such approaches to metalloenzymes in combination with mechanistic studies are often overlooked. In this review, we summarize our recent structural and kinetic studies on a set of three heme peroxidases found in the bacterium *Streptomyces lividans* that each belong to the dye decolourizing peroxidase (DyP) superfamily. Kinetically, each of these DyPs has a distinct reactivity with hydrogen peroxide. Through a combination of low dose synchrotron X-ray crystallography and zero dose serial femtosecond X-ray crystallography using an X-ray free electron laser (XFEL), high-resolution structures with unambiguous redox state assignment of the ferric and ferryl (Fe^IV^ = O) heme species have been obtained. Experiments using stopped-flow kinetics, solvent-isotope exchange and site-directed mutagenesis with this set of redox state validated DyP structures have provided the first comprehensive kinetic and structural framework for how DyPs can modulate their distal heme pocket Asp/Arg dyad to use either the Asp or the Arg to facilitate proton transfer and rate enhancement of peroxide heterolysis.

**Graphic abstract:**

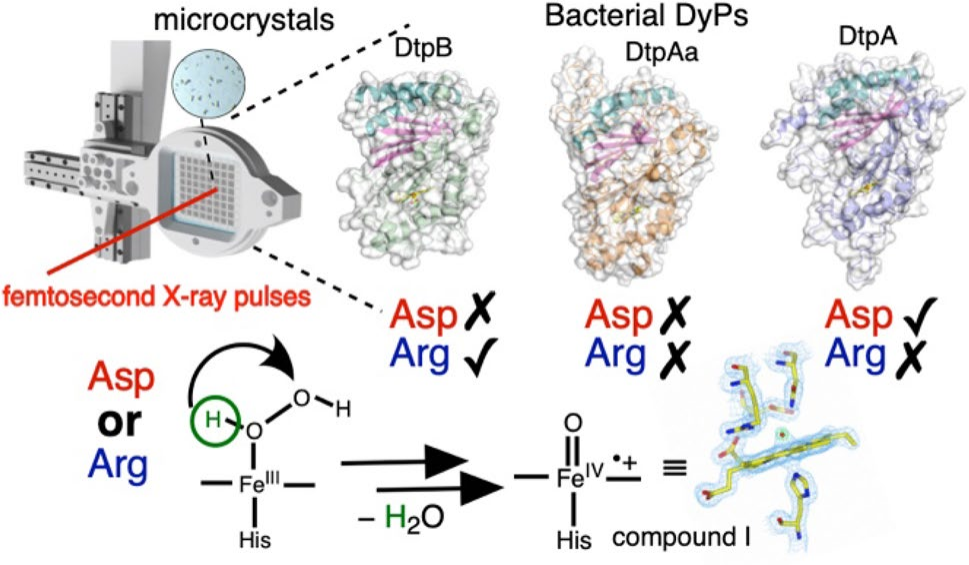

## Introduction

Dye-decolorizing peroxidases (DyPs; EC 1.11.1.19) are the most recently discovered member of the histidine-heme ligated peroxidase superfamily [1]. They are widespread in bacterial and fungal genomes [2] and represent a distinct heme peroxidase structural family that possess a dimeric α + β barrel fold (SCOP 3,000,089, InterPro Pfam CL0032) capable of binding a single *b*-type heme [3, 4]. DyPs were initially divided into four phylogenetic classes; types A, B, C and D [5], with types C and D later found to be one phylogenetic class and now referred to as C/D-type. The A- and B-types are exclusively found in bacteria whilst the C/D-types are found in bacteria and fungi [5]. Historically, their name derives from the discovery that a C/D-type displayed an ability to oxidise recalcitrant anthraquinone and/or azo-dyes used in the textile industry [1]. However, it has become apparent that not all DyPs possess an ability to oxidise these redox dyes and thus the initial naming of this family of heme peroxidases is in hindsight rather unfortunate [6]. In fact, the physiological function of DyPs is almost completely unknown, with only a handful of examples illustrating a potential role of fungal C/D-types in lignin oxidation [7, 8] and in the degradation of an antifungal anthraquinone compound [9]. Bacterial DyPs are also associated with encapsulin systems [10–12]. These proteinaceous nanocompartments are assembled into icosahedral hollow capsids by one type of shell protein that serve to encapsulate cargo proteins [10, 13, 14]. A recent cryo-EM study has revealed that oligomeric DyP assemblies (dodecamers) can pack inside a capsid [15].

All DyPs react with hydrogen peroxide to form high-valent heme species, commonly referred to as ferryl heme [16–18], essential for the oxidative chemistry that results in electron transfer from substrates. Although their physiological substrates remain unknown, many DyPs, in addition to reacting with synthetic dyes, display a wide substrate specificity with artificial electron donors such as ABTS, guaiacol and ferrocyanide [19]. The initial ferryl species formed on reaction with peroxide is a two-electron oxidised Fe^IV^-oxo derivative carrying a porphyrin π-cation radical and has an electronic absorbance spectrum analogous to the Compound I species first identified in horse radish peroxidase (HRP) [20, 21]. Following the one-electron reduction of the porphyrin π-cation radical, a second ferryl species is formed, that has spectral properties analogous to HRP Compound II [20, 21], with a further one-electron reduction returning the heme to the ferric resting state. Concomitant with this peroxide-induced cycle is the coproduction of two water molecules and oxidised substrates [20–23].

In the well-studied nonmammalian peroxidases such as HRP, ascorbate peroxidase (APX) and yeast cytochrome c peroxidase (CcP), a His-Arg dyad is present in the distal heme pocket (*i.e.* the ‘face’ of the heme that binds peroxide), with the His assigned a catalytic role in facilitating proton movement involved in Compound I formation [24–27]. It is now recognised that the distal pocket His is hydrogen bonded (H-bonded) to a water molecule, to create a water-His unit that is energetically more favoured for moving a proton from the O^α^ atom of the Fe^III^-O_2_H_2_ complex [28], to generate an anionic precursor to Compound I referred to as Compound 0 (Fe^III^-O^α^-O^β^H) [29, 30]. The H^α^ proton is then transferred from the distal water-His unit to the O^β^ atom of Compound 0 to form Fe^III^-O-OH_2_ that promotes the heterolytic cleavage of the O–O bond to form Compound I and the coproduction of a water molecule [24, 28]. In DyPs, the distal pocket His residue is replaced by an Asp, which is part of the GXXDG motif, unique to DyP members [3, 31]. Whereas in nonmammalian peroxidases the distal heme Arg is indirectly involved in Compound I formation [27, 32], studies on a handful of DyPs have provided evidence to indicate that the Arg is directly involved, implying a catalytic role as a proton acceptor and donor [33–35]. In other DyPs the Asp has been reported to act as the acid–base catalyst [3, 36–39]. Thus, it has become clear that there is mechanistic variation in Compound I formation amongst DyPs and the question of why Asp or Arg is selected to facilitate proton transfer and rate enhancement of O–O fission amongst DyP members remains unanswered.

## A holistic approach to studying mechanistic features of DyP members

In the soil-dwelling Gram-positive bacterium, *Streptomyces lividans*, three DyPs are present. Based on sequence analysis, two are classed as A-types, each possessing a signal sequence that locates them in the extracytoplasmic environment. The third is a B-type, which from the absence of a signal sequence remains located in the cytoplasm. The physiological substrates for these *S. lividans* DyPs remain unknown, but one of the A-types (Dye-type peroxidase A; DtpA) has been implicated in a copper trafficking pathway that regulates a morphological development step in the life-cycle of *S. lividans* [40]. Despite the presence of multiple DyP encoding genes in many bacterial and fungal genomes, only a handful of rather fractured studies enable some comparisons of the structural and kinetic properties of DyPs from the same organism [41–44]. We have, therefore, focused on a holistic approach using the three *S. lividans* DyPs to decipher mechanistic intricacies of Compound I formation. This mini review highlights recent structural and kinetic approaches taken to investigate these *S. lividans* DyPs and presents a new framework for understanding different mechanisms of Compound I formation adopted by DyPs. It also focuses on the need to pay careful attention to the often insidious effects of X-ray radiation, used in crystallographic data collection, on the metal site structure, so as to avoid misinterpretation and misassignment of structures to particular redox states.

## Validation of redox state in X-ray structures is essential for deciphering mechanism

With the advent of high brilliance synchrotron radiation sources and their high-throughput data collection capabilities, X-ray crystal structure determination of proteins and enzymes has become a relatively routine and increasingly automated process, with the often high-resolution structures obtained used to assist in the interpretation of mechanism. It is now recognised that if the appropriate experimental safeguards in the collection of X-ray diffraction data have not been put in place, there is a danger of a mismatch between the determined structures and mechanism [45]. This is because the high-intensity X-rays generated at synchrotron sources are partially absorbed by protein crystals, all of which have a high water content. Radiolysis of this water produces multiple radical species including a large number of solvated photoelectrons that can cause not only global radiation damage (*i.e.* degradation of the crystalline order and hence diffracting properties of the crystal, which to a certain extant can be countered by cryo-cooling the crystals to 100 K), but also site-specific radiation damage [46–48]. This site-specific radiation damage can, in the case of metalloenzymes, reduce redox active metals via solvated photoelectrons and hence change the coordination geometry and the coupled protein structure. Thus, site-specific radiation damage is a particular concern for the determination of heme-Fe redox states in peroxidases [45, 49], with the resting state Fe^III^ and high-valent Fe^IV^ species both exquisitely susceptible to site-specific radiation damage. Such damage can occur at X-ray doses at least three orders of magnitude lower than the 20–30 MGy range, which is an acceptable absorbed dose limit for global radiation under cryo-cooled conditions [50, 51]. The very low doses required for redox state changes essentially rule out the effective determination of non-damaged states from single crystals at either cryogenic or room temperature. Lowering the X-ray flux, for example by using an in-house diffractometer or a very attenuated synchrotron beam would require either a greatly increased data collection time (thereby incurring a similar dose as using a more intense source for a shorter time) or accepting a much lower resolution dataset which may not reveal the details of the metal centre environment. Therefore, to capture intact (*i.e.* free of radiation-induced damage) or near-intact structural data from radiation-sensitive Fe^III^ and Fe^IV^ heme species using X-ray crystallography, two general approaches have been developed (i) low-dose composite (*i.e.* multi-crystal) structures coupled with single crystal spectroscopy to validate redox state before and after X-ray exposure (near-intact) [35, 39, 52–55] and (ii) the use of X-ray free electron lasers (XFELs, zero effective dose) [35, 56, 57].

The first DyP X-ray structure was reported in 2007 [3], and in the subsequent years over 50 DyP structures have been deposited in the Protein Data Bank (PDB). Table 1 provides a summary of the deposited DyP structures. Except for the *S. lividans* DyP structures, and several recently reported structures from the B-type *Klebsiella pneumoniae* DyP [45], none of the deposited DyP structures report a validated redox state or mitigating data collection strategies. Pfanzagl et al*.* [45] have reported that an absorbed dose of ~ 40 kGy for cryo-cooled heme protein crystals is sufficient to cause a 50% reduction of the heme iron redox state independent of oxidation state (*i.e.* Fe^III^ or Fe^IV^). Therefore, it can be safely assumed, with the exceptions indicated in Table 1, that all the DyP structures deposited in the PDB are representative of either mixed heme oxidation species or the Fe^II^-heme state. Nevertheless, despite this limitation, many of the deposited structures have been used in combination with spectroscopic and kinetic data to inform on mechanistic features associated with the formation of transient Fe^IV^-oxo intermediates starting from the Fe^III^-heme resting state. Whilst the overarching mechanistic conclusions may be predominantly correct, details of subtle local structural changes and perturbations to solvent networks in non-validated structures may be missed, which can have significant bearing for a complete mechanistic understanding. This is particularly the case for distal pocket water molecules, which up until recently have never been discussed in the mechanism of Compound I formation in DyPs. Two separate reports on X-ray dose-dependent effects on cryo-cooled DyP crystals highlight such local changes to (i) side chain positions of distal heme pocket residues, including the Asp and (ii) distal pocket H-bonded water networks, between the Fe^III^-heme and Fe^II^-heme states [45, 49]. These changes are significant and would undoubtedly confound mechanism assignment, particularly for DtpA [39, 49, 55]. Shrestha et al*.* [58] have compared side chain positions of distal heme pocket residues and H-bonding patterns of distal water molecules between several B-type DyP X-ray crystal structures (3QNS, 5VJ0 and 6FKS, Table 1) to rationalise kinetic data for Compound I formation from the ferric state. These structures can reasonably be assumed to be in either a mixed Fe^III^/Fe^II^-heme state or an Fe^II^-heme state, which in the case of the 6FKS structure has subsequently been shown to be the ferrous state [45]. Furthermore, previously validated structural work on a B-type DyP [35] for which the distal Arg is demonstrated to be important for proton movement in Compound I formation (discussed further below) is overlooked in the investigator’s mechanism [58].

**Table 1:** A summary of the X-ray crystal structures of DyPs deposited in the PDB

Organism	Class	Mutation	PDB code and resolution	Comment
Structures where a redox state is validated or a dose reported				
*Streptomyces lividans*	A	WT	5MJH (1.45 Å), 5MAP (1.49 Å) [49]	Oxyferrous forms
		WT	6GZW (1.41 Å) [55]	Ferric form (see Table 2)
	A (Aa)	WT	6TB8 (1.80 Å) [39]	Ferric form (see Table 2)
		WT	6I43 (1.88 Å) [57]	Ferric form (see Table 2)
		WT	6I7C (1.88 Å) [64]	SFX, ferric form with imidazole bound at 6th heme coordination position
		WT	8I8P, 6Q3E, 6Q3D, 6I8K, 6I8I, 6I7Z, 6I8O, 6Q34, 6I8Q, 6I8E, 6Q31, 6I8J, 6IBN (1.70–193 Å) [57]	Ferric dose series (32.8 kGy), serial synchrotron
	B	WT	6YRC (1.99 Å), 6YRJ (1.85 Å) [35]	Ferric form (see Table 2)
		WT	6YR4 (1.85 Å), 6YRD (1.75 Å) [35]	Ferryl Compound I (see Table 2)
*Klebsiella pneumoniae*	B	WT	6RQY, 6RR1, 6RR4, 6RR5, 6RR6, 6RR8 (1.90 Å) [45]	Ferric dose series (2.15 – 53.6 kGy)
		WT	6RPE (1.80 Å), 6RPD (1.52 Å) [45]	Ferric with cyanide bound at 6th heme position, doses 2.3 kGy and 1590 kGy
Structures where the redox state is *not* validated and no dose reported				
*Cellulomonas bogoriensis*	A	WT	6QZO (2.40 Å) [103]	A natural substitution of the distal Asp with a Glu
*Thermomonospora curvata*	A	WT	5JXU (1.75 Å) [104]	
*Escherichia coli* O157	A	WT	3O72 (1.95 Å) [42]	
*Streptomyces coelicolor*	A	WT	4GT2 (1.80 Å)^a^	A-type homologs with 99% sequence identity to *S. lividans*
	A (Aa)	WT	4GRC (2.00 Å)^a^	
*Bacillus subtilis*	A	WT	6KMN (2.44 Å) [105]	6KMM has HEPES bound at a surface site
		WT	6KMM (1.93 Å) [105]	
*Thermobifida fusca*	A	WT	5FWV (1.80 Å) [106]	
*Thermobifida cellulosilytica*	A	WT	4GS1 (1.70 Å)^a^	
*Rhodococcus jostii*	B	WT	3QNR (2.25 Å), 3QNS (1.40 Å) [43]	Forms a hexamer assembly and possess a C-terminal motif to target it to the encapsulin nanocompartment [11]
		N246H	3VEF (2.64 Å) [33]	4HOV has manganese bound in a pocket
		N246A	3VEE (2.40 Å), 4HOV (2.20 Å) [33]	
		R244L	3VEG (2.35 Å) [33]	
		D153H	3VED (2.50 Å) [33]	
		D153A	3VEC (2.60 Å) [33]	
*Klebsiella pneumoniae*	B	WT	6FKS (1.60 Å) [71]	
		D143A	6FL2 (1.27 Å) [71]	
		R232A	6FKT (1.86 Å) [71]	
		D143A/R232A	6FIY (1.09 Å) [71]	
*Shewanella oneidensis*	B	WT	2HAG (2.75 Å) [107], 2IIZ (2.30 Å) [108]	No heme and heme bound
*Bacteroides thetaiotaomicron*	B	WT	2GVK (1.60 Å) [107]	No heme; hexamer assembly
*Vibrio cholerae*	B	WT	5DE0 (2.24 Å) [109]	
*Escherichia coli* O157	B	WT	5GT2 (2.09 Å) [44]	
*Enterobacter lignolyticus*	B	WT	5VJ0 (1.30 Å) [37]	
*Streptomyces coelicolor*	B	WT	4GU7 (3.10 Å)^a^	99% sequence identity to *S. lividans*
*Dictyostelium discoideum*	B	WT	7O9J (1.70 Å) [110]	
		WT	7O9L (1.85 Å) [110]	Cyanide bound to heme iron
		WT	7ODZ (1.60 Å) [110]	Veratryl alcohol bound on surface
*Amycolatopsis sp.*	C/D	WT	4G2C (2.25 Å) [111]	Manganese binding pocket
*Nostoc sp.*	C/D	D204H	5C2I (1.89 Å) [112]	Tetramer assembly formed from Cys224-Cys224 disulfide-linked dimers
*Bjerkandera adusta*	C/D	WT	2D3Q (2.80 Å) [3], 3AFV (1.40 Å) [113]	2D3Q first crystal structure of a DyP
		WT	3MM2 (1.45 Å) [113]	Cyanide bound to heme
		WT	3VXI (1.50 Å)^a^	Ascorbate bound at a surface site
		WT	3VXJ (1.39 Å)^a^	2,6-dimethoxyphenol bound at a surface site
		D171N	3MM1 (1.42 Å), 3MM3 (1.40 Å) [113]	Cyanide bound to heme iron in 3MM3
*Auricularia auricula-judae*	C/D	WT	4AU9 (2.10 Å) [114], 4W7J (1.79 Å) [115]	5AG1 has a δ-meso-nitrated heme
		WT	5AG1 (1.85 Å)^a^	
		WT	5AG0 (1.75 Å)^a^	
		WT	4UZI (2.20 Å) [116]	4UZI has imidazole at the 6th heme coordination position and HEPES bound at a surface site
		Y47S	4W7K (1.05 Å) [115]	
		D168N	4W7L (1.05 Å) [115]	
		W377S	4W7M (1.15 Å) [115]	
		Y147S/W377S	4W7N (1.40 Å) [115]	
		Y147S/G169L/W377S	4W7O (1.20 Å) [115]	
		F359G	5IKD (1.11 Å) [117]	5IKD highly stereoselective in oxidising phenyl-sulides (S-enantiomer) 5IKG mixed S- and R-enantiomers
		L357G	5IKG (1.95 Å) [117]	
*Pleurotus ostreatus*	C/D	F194Y	6FSK (1. 56 Å) [118]	6FSK MES bound to a surface site; possess a non-canonical manganese binding site
		F194W	6FSL (2.50 Å)^a^	

^a^No associated publication

s

## Approaches used to obtain Fe^III^-heme resting state structures of *S. lividans* DyPs

**Fig. 1:**
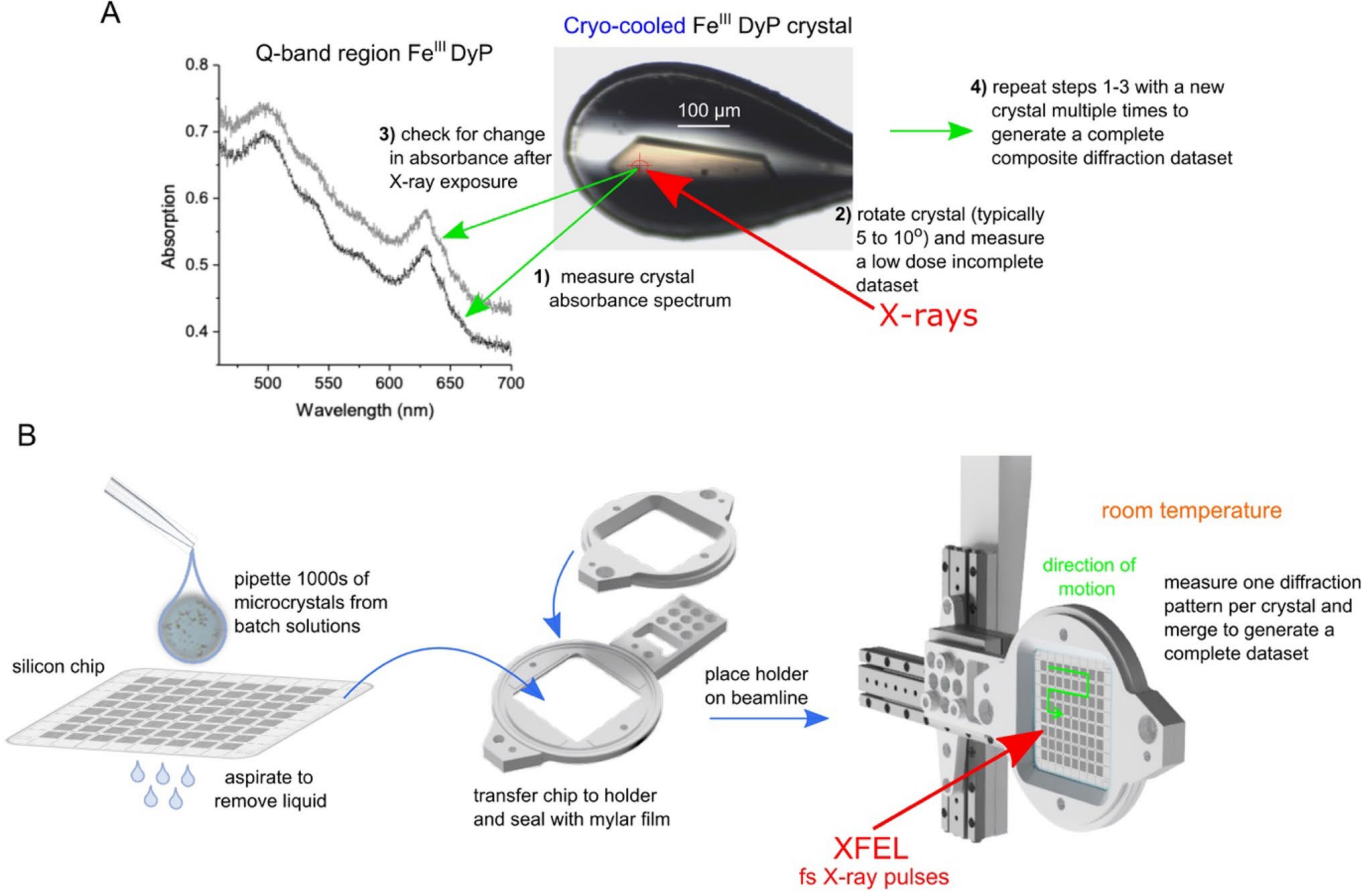
Approaches used to obtain low-dose (**A**) or zero-dose (**B**) X-ray crystal structures of the *S. lividans* DyPs reported in Table 2. In (**A**) composite synchrotron X-ray data are collected using multiple cryo-cooled crystals with microspectrophotometry used to monitor the heme oxidation state. **B** The chip delivery system for SFX crystallography using microcrystals at room temperature

The starting point for our mechanistic investigations into the three *S. lividans* DyPs has been to obtain intact or near-intact structures of the resting state Fe^III^-heme enzyme. To achieve this goal, several X-ray approaches have been successfully applied. Figure 1 summarises the strategies that have been used and Table 2 the pertinent experimental and data processing parameters for the structures determined and deposited in the PDB. For Fe^III^-DtpA, a single crystal, low-dose, helical data collection strategy was employed at 100 K with the redox state of the DtpA crystal monitored along the X-ray exposed pathway using in situ UV–Vis microspectrophotometry [55]. The Q-band region of the electronic absorbance spectrum of peroxidases has several prominent peaks that allow the Fe^III^- and Fe^II^-heme redox states to be readily distinguished (Fig. 1A). Composite crystallographic datasets using a multi-crystal strategy at 100 K have been collected for DtpAa and DtpB (Table 2) [35, 39], making use of in situ microspectrophotometry to monitor the Q-band region of the absorbance spectrum with spectra recorded pre- and post-X-ray exposure of each crystal (Fig. 1A). This allowed the extent of any photoreduction to be assessed and crystals with an unacceptably high level of reduction to be excluded from the final composite dataset. For both the helical and composite datasets, the absorbed dose was in the low tens of kGy range, with the respective values reported in Table 2. It is noted that reported doses in Table 2 are all less than the typical 40 kGy limit determined to induce 50% reduction of Fe^III^-heme [45], but are nevertheless sufficient to cause site-specific radiation damage. However, in combination with the comparison of the before and after microspectrophotometry data, the structures can be considered as near-intact resting state Fe^III^-heme. Notably, exposure to higher doses resulted in clear reduction of the crystals, leading to spectral properties consistent with the ferrous form.

**Table 2:** Summary of strategies used to determine the redox state validated X-ray structures of *S. lividans* DyPs along with selected experimental and data processing parameters

	Fe^III^-DtpA	Fe^III^-DtpAa	Fe^III^-DtpAa	Fe^III^-DtpB	Fe^III^-DtpB	Fe^IV^-DtpB	Fe^IV^-DtpB
X-ray source	ESRF	SLS	SACLA	SLS	SACLA	SLS	SACLA
Temperature (K)	100	100	298	100	298	100	298
Number of crystals	1	13	72,615^a^	21	26,223	13	57,909
Collection mode	helical along a translation of 300 μm	composite, 20° wedges, 0.1° oscillation	SFX, pulse length 10 fs, repetition rate 30 Hz, 11 chips used	composite, 10° wedges, 0.1° oscillation	SFX, pulse length 10 fs, repetition rate 30 Hz, 4 chips used	composite, 8° wedges, 0.1° oscillation	SFX, pulse length 10 fs, repetition rate 30 Hz, 5 chips used
Microspectrophotometry	Yes	Yes	No	Yes	No	Yes	No
Effective dose (kGy)	12.0	17.0–21.7	0	11.4	0	11.3	0
Space group	P2_1_	P2_1_	P2_1_	P2_1_2_1_2_1_	P2_1_2_1_2_1_	P2_1_2_1_2_1_	P2_1_2_1_2_1_
Unit cell dimensions (Å)	*a* = 60.1, *b* = 71.0, *c* = 78.1	*a* = 71.4, *b* = 67.6, *c* = 72.9	*a* = 72.7, *b* = 68.2, *c* = 74.6	*a* = 85.8, *b* = 120.3, *c* = 196.0	*a* = 86.7, *b* = 121.6, *c* = 199.0	*a* = 85.4, *b* = 119.9, *c* = 194.2	*a* = 86.3, *b* = 121.1, *c* = 198.5
Resolution (Å)	1.41	1.80	1.88	1.99	1.85	1.85	1.75
PDB identifier	6GZW	6TB8	6I43	6YRC	6YRJ	6YR4	6YRD

^a^number of indexed images used, the number of crystals will be less

To eliminate site-specific radiation damage, femtosecond-duration pulses from an XFEL source can be used, which allow the collection of diffraction patterns before the onset of radiation damage [59–63]. While the X-ray dose is tremendously high in such experiments, the extremely short duration of the pulses means that there is insufficient time under typical experimental conditions (pulse durations of ~ 10 fs or less) for any radiation damage to be manifest in the observed structure. This principle is termed ‘diffraction-before-destruction’ [59], with the approach called serial femtosecond crystallography (SFX) and requires the presentation of a new crystal for each pulse. Thus, to obtain a protein structure using SFX, thousands of single-crystal diffraction patterns must be collected from microcrystals (≤ 50 μm) efficiently presented to the X-ray beam. An approach that we have found successful for determining zero dose structures of DyPs is the use of silicon fixed-target chips containing 25,600 apertures that are used to trap the microcrystals which are applied in suspensions of between 100 and 200 μl (Fig. 1B) [35, 57, 64]. The chips are translated within the interval between XFEL pulses stopping at the centre of each aperture allowing for the crystal to be exposed only once to the X-ray pulse, before moving to the next position during the pulse interval (Fig. 1B). An advantage of this and other serial delivery systems is that the SFX data can be collected at room temperature and, therefore, affords the opportunity to acquire structures and observe chemistries that are not frozen out, which can be the case with data collection at 100 K [65]. Using this chip-based SFX approach we have been able to determine zero dose structures of DtpAa and DtpB in which the Fe^III^-heme state is considered pristine (Table 2) [35, 57].

## Tertiary structure comparison of the three *S. lividans* DyPs

**Fig. 2:**
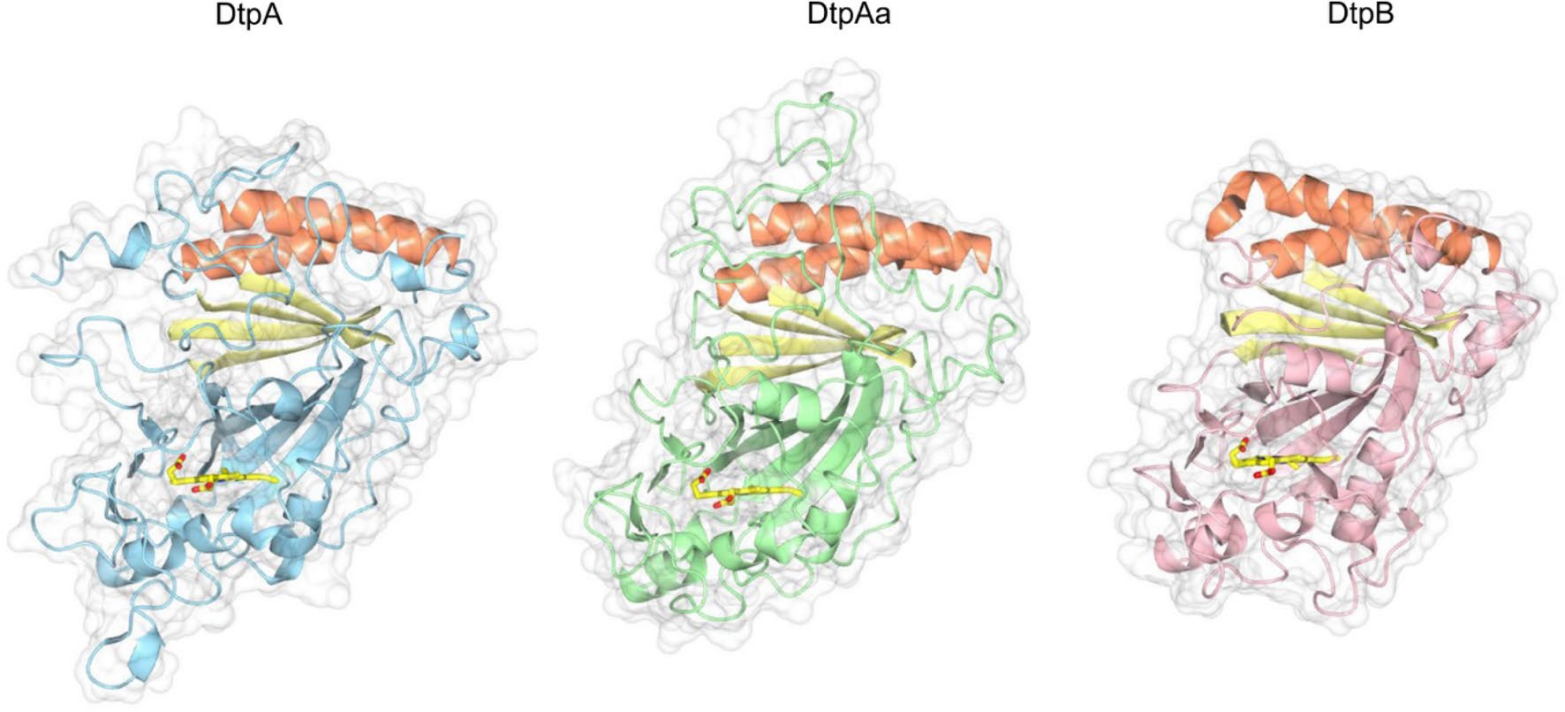
Tertiary structures of the three *S. lividans* DyPs in their Fe^III^-heme redox state determined using X-ray methods at 100 K as outlined in Table 2. One of the α + β barrel fold in each structure is highlighted in orange (α) and yellow (β) with the heme shown in sticks. PDB codes used are 6G2W (DtpA); 6TB8 (DtpAa); 6YRC (DtpB)

A comparison of the tertiary structures of the three *S. lividans* DyPs determined using the X-ray approaches summarised in Table 2 are depicted in Fig. 2. The structural homology of the dimeric α + β barrel fold is conserved, with changes in helical content and loop insertions/deletions contributing to the variation in structural shape and molecular weight (Fig. 2). In all cases the *b*-type heme is located in the C-terminal domain with the heme-Fe participating in proximal coordination via the N^δ^ atom of a His residue. The N^ε^ atom of the proximal His ligand is H-bonded to the O^δ2^ atom of a conserved Asp, an interaction that imparts significant imidazolate character in His-heme ligated peroxidases resulting in increased electron-donating ability [23].

## Distinct distal heme pocket sites in the resting state structures of the *S. lividans* DyPs

By determining the pristine and near-intact heme-Fe^III^ structures for each *S. lividans* DyP, a true comparison of the distal heme environment, where the chemistry with peroxide takes place, is now possible [35, 39, 55, 57].

### DtpA and DtpAa

**Fig. 3:**
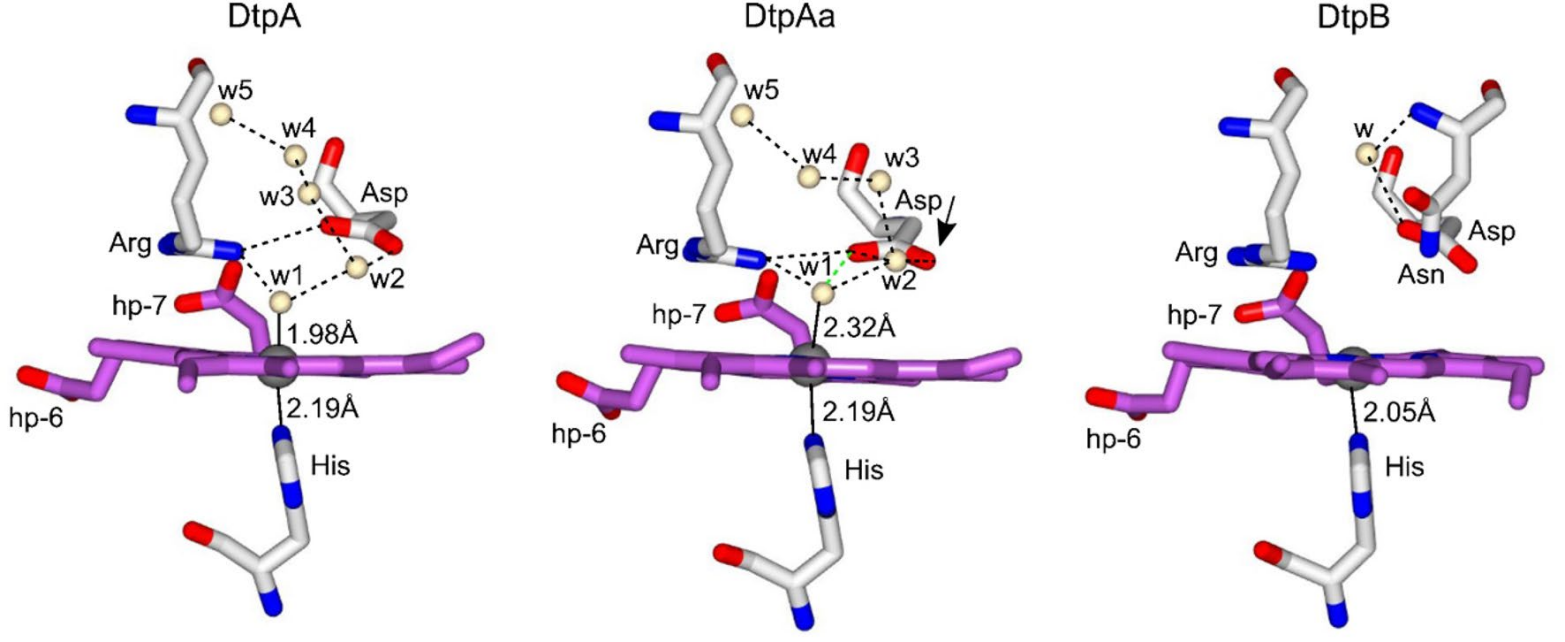
Comparison of the validated Fe^III^-heme sites in each of the DyPs from *S. lividans*. Water molecules (w) are depicted as small cream spheres, hp refers to heme propionate groups and H-bond interactions are shown as dashed lines. In DtpAa, the green dashed line indicates the additional H-bond to w1, absent in DtpA, which result from the positional change of the distal Asp residue as indicated by the arrow. PDB codes used are 6G2W (DtpA); 6TB8 (DtpAa); 6YRJ (DtpB)

For the two A-type enzymes, the distal heme pocket is dominated by a well-ordered network of H-bonded water molecules (crystallographic B-factors range between 10 and 20 Å^2^) that enable communication between the heme-Fe^III^ and the distal Asp/Arg dyad (Fig. 3) [39, 55]. It is important to note that the stereochemistry of the DtpAa heme-Fe^III^ environment is identical between the composite 100 K structure and the XFEL room temperature structure, including the positions of the H-bonded water molecules [39, 57]. This indicates that despite the overall absorbed dose for the 100 K structure (Table 2) the approach of using composite datasets from multiple low-dose wedges and microspectrophotometry nullifies significant specific radiation damage as evidenced from comparison with the zero dose XFEL structure [39, 57].

In both the A-type structures a water molecule is found close enough to the heme-Fe^III^ for a bonding interaction to take place and thus the heme is considered to exist in a hexacoordinate state (Fig. 3) [39, 55, 57]. The electronic absorption (solution and crystal) and EPR spectra are consistent with a high-spin ferric heme, and thus the water ligand is not sufficient to drive the heme to a low-spin ferric state [39, 55]. On closer inspection of the heme-Fe^III^ environment between the two enzymes, subtle differences emerge. Of significance is a side-chain positional shift of the distal Asp residue towards the heme in DtpAa (Fig. 3). This change, observed in both the 100K composite structure and room temperature XFEL structure, permits a H-bonding interaction with the Fe^III^-heme coordinated water (w1) [39, 57], an interaction which is absent in DtpA [66], and may coincide with the longer w1-Fe^III^ bond-length observed in DtpAa (Fig. 3) [39, 57]. In contrast, the distal Arg position and H-bonding pattern, as indicated in Fig. 3, remains the same between the two A-type structures [39, 55, 57]. Therefore, having confidence that the structures represent the true stereochemistry of the Fe^III^-heme site, the subtle side-chain positional change and altered H-bonding water pattern for the distal Asp residue between the two A-type homologues in *S. lividans* can be considered significant, rather than being an artefact caused by differing oxidation states of the enzymes [39, 55, 57].

### DtpB

For DtpB the stereochemistry of the distal heme-Fe^III^ environment between the composite 100 K and room temperature XFEL structures is again identical [35]. Notably both structures reveal an absence of resident water molecules in the distal heme pocket [35]. Thus, in contrast to the ‘wet’ distal heme pocket found for the A-type enzymes [39, 55], in DtpB the pocket is ‘dry’ (Fig. 3) [35]. The heme in DtpB is, therefore, pentacoordinate with the Fe^III^ atom sitting slightly out of the porphyrin plane towards the proximal His ligand, creating a shorter Fe^III^-N^δ^-His bond than observed in either DtpA or DtpAa (Fig. 3). Thus, the communication between the distal pocket Asp/Arg dyad and the heme-Fe^III^ that exists through the H-bonded water network in DtpA and DtpAa is lost in DtpB [35].

An Asn side chain protrudes into the distal heme environment in DtpB, with its amide group occupying the spatial position where in the A-type enzymes a water molecule (w2) is located and H-bonded to the distal Asp (Fig. 3) [35, 39, 57]. It has been suggested that the presence of the Asn causes a steric impediment to the formation of a distal water network in DtpB [35]. However, as will be discussed further, the absence of a w2-Asp interaction has consequences for whether Asp or Arg is selected to facilitate proton transfer and rate enhancement of Compound I formation [39].

## Peroxide access channels

**Fig. 4:**
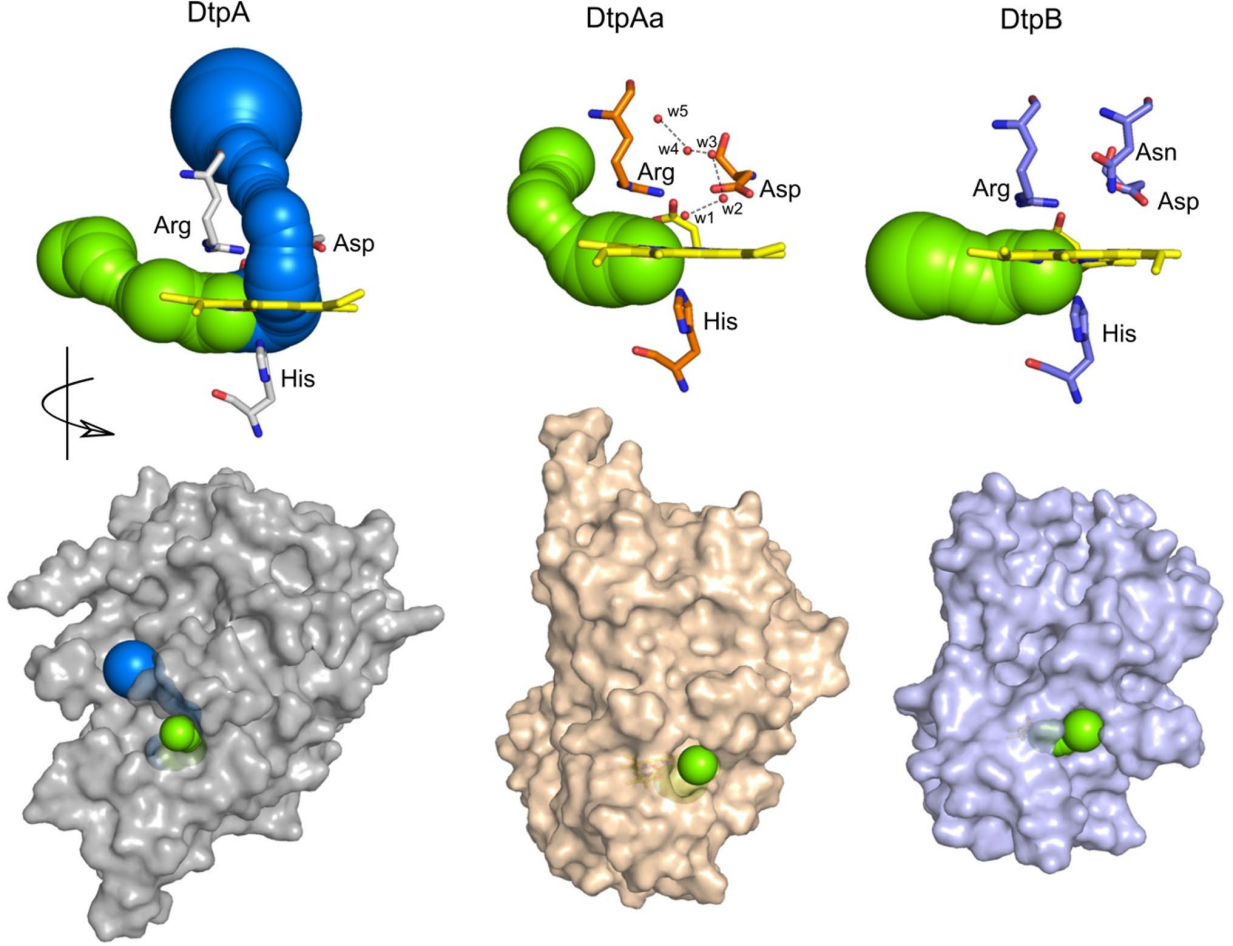
Putative peroxide access channels in the three *S. lividans* DyPs. CAVER 3.0 was used to calculate channels originating from the heme-Fe and connecting to a solvent surface opening using the following settings: minimum probe radius = 1.2; shell depth = 8; shell radius = 7; clustering threshold = 4. For DtpA two predominant pathways were identified (blue and green), whereas for DtpAa and DtpB only one pathway was identified. The surface representations of the respective DyPs indicate where the entry to the channels reside. The green channel is common to all three DyPs and enters via the γ heme edge, between the two propionate groups. In DtpA the blue channel enters into the distal side of the heme. Both channels are lined with polar residues and contain H-bonded waters. PDB codes used are 6G2W (DtpA); 6TB8 (DtpAa); 6YRJ (DtpB)

A question that arises from the resting state Fe^III^-heme structures is whether there are any obvious structural features, other than the possible presence of the Asn side chain in the distal pocket of DtpB, that can account for a ‘dry’ versus ‘wet’ distal heme site. Surprisingly, an inverse correlation of heme accessible surface area (ASA) is found in the order DtpB (37.4 Å^2^) > DtpAa (11.8 Å^2^) > DtpA (11.2 Å^2^), clearly indicating a more solvent insulated heme in the A-type enzymes [35]. Insight into how water or peroxide can enter into the heme environment can be investigated using CAVER 3.0 [67] that can calculate the location of putative access channels connecting the heme to the bulk solvent. DtpA has two such channels (Fig. 4). One runs from a surface opening down into the distal heme pocket, essentially following the route of the H-bonded water network (w1 to w5, Fig. 3) and the second channel protruding from the γ side of the heme, adjacent to the heme propionate-6 group (Fig. 4). This latter channel is also occupied by water molecules that form a continuous H-bonding network from the bulk solvent to the Nε atom of the distal Arg residue [55]. DtpAa and DtpB on the other hand possess only one surface access channel to the heme [35, 39], which is identical in location to the second channel in DtpA [55]. It is noticeable that the surface entrance diameter for this channel in DtpB is larger than in either DtpA or DtpAa (Fig. 4). Thus, based on structural analysis, it is not clear why DtpA and DtpAa accommodate resident waters in the distal heme site as opposed to DtpB, which despite a more solvent accessible heme, favours a ‘dry’ distal heme site. This begs the question of whether it is the ease of solvent egress in DtpB which accounts for increased exposure to solvent of the heme site.

## An over-view of the transient kinetics of Compound I formation

**Fig. 5:**
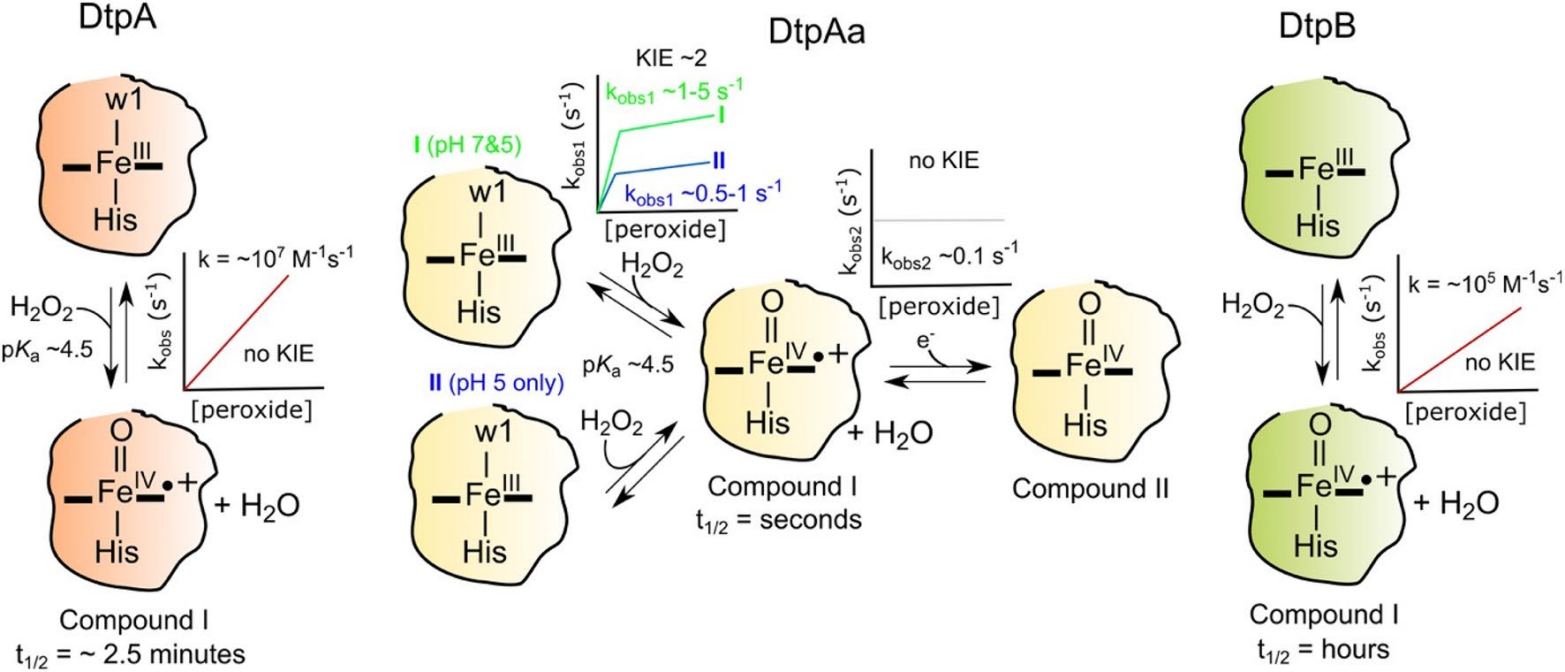
A cartoon summary of the stopped-flow reaction kinetics reported for the *S. lividans* DyPs upon reacting with peroxide. For each DyP the chemistry that occurs within the heme pocket as determined from absorbance spectrum changes on mixing with peroxide is depicted, along with illustrative plots of *k*_obs_ versus peroxide concentration. The DtpA and DtpAa Fe^III^-heme states are illustrated with a coordinating water molecule (w1) as corroborated by the X-ray crystal structures. For DtpAa, two Fe^III^-heme species exist at pH 5, species I and II, with species I displaying the same kinetics at pH 7, where only one form exists. All kinetic experiments were conducted at 25 °C

Understanding the mechanism of formation and chemical nature of Compound I has been a focal point of peroxidase chemistry for many years [22, 23]. Stopped-flow absorption spectroscopy is a commonly used approach to monitor Compound I formation by mixing the resting state ferric enzyme with peroxide. The subsequent reaction results in spectral changes in the Soret and Q-band region of the electronic absorption spectrum that, for many peroxidases, allow Compound I and Compound II to be distinguished and identified [22]. Figure 5 provides an illustrative summary of the salient kinetic features of Compound I formation reported for each of the *S. lividans* DyPs upon mixing with peroxide using stopped-flow absorption spectroscopy [19, 35, 39].

### DtpA and DtpB

For DtpA and DtpB the kinetic behaviour follows a single spectral transition, resulting in a spectrum with wavelength features in the Soret and Q-band region typical for a Fe^IV^-oxo species carrying a porphyrin π-cation radical, *i.e.* Compound I [19, 35]. A linear dependence on the observed pseudo-first order rate constants (*k*_obs_) with increasing peroxide concentration allows a second-order rate constant for Compound I formation (*k*_cmpdI_) to be determined (Fig. 5). Notably, DtpA possesses a *k*_cmpdI_ in the region of 10^7^ M^−1^ s^−1^ [19], similar to that of HRP [68, 69], and is thus a highly reactive peroxidase. In contrast, DtpB exhibits a *k*_cmpdI_ in the region of 10^5^ M^−1^ s^−1^, two-orders of magnitude lower than DtpA [35]. Nevertheless, the linear dependence on *k*_obs_ with increasing peroxide concentration for both DtpA and DtpB, indicates binding of peroxide to the Fe^III^-heme is rate limiting for Compound I formation. Once Compound I is formed its life-time in the absence of an exogenous electron donor varies between DtpA and DtpB [19, 35]. For DtpA a half-time (t_1/2_) of ~ 2.5 min is reported [19], with EPR spectroscopy revealing that the electron to fill the porphyrin π-cation ‘hole’ is acquired through the oxidation of a Tyr residue (Tyr374) to generate a Fe^IV^ = O Tyr^•^ species *(i.e.* internal electron transfer) [55]. By contrast, Compound I in DtpB is stable for hours, with EPR spectroscopy reporting a signal at *g* = 2 with a strongly asymmetric line-shape that does not change intensity and shows no saturation with microwave power, typical of a Compound I species carrying a porphyrin π-cation radical [35]. Thus, Compound I is more reactive in DtpA than in DtpB.

### DtpAa

For DtpAa the kinetics of Compound I formation are significantly different from those described above (Fig. 5). The spectral transitions observed upon mixing with peroxide reveal the presence of an intermediate species, and on fitting to a sequential mechanism (*i.e. a* → *b* → *c*) this intermediate species (*b*) is shown to have spectral features associated with Compound I and the end point spectrum (*c*) consistent with a Compound II species [39]. Thus, unlike in DtpA and DtpB, where a single spectral transition to Compound I is observed, in DtpAa, Compound I is formed but decays (in the absence of exogenous electron donor) to a Compound II species (Fig. 5). Notably, a non-linear dependence of *k*_obs_ for Compound I formation with increasing peroxide concentration is observed (Fig. 5) [39]. Such a reaction rate profile has been suggested to mean that at low peroxide concentration the rate limit is peroxide binding to the Fe^III^-heme as evidenced by the steep increase in *k*_obs1_ values, whilst at higher peroxide concentration a second rate limit takes over (Fig. 5) [39]. To further complicate the DtpAa kinetics, both stopped-flow spectral analysis and EPR spectroscopy reveal the presence of two Fe^III^-heme species at pH 5, but only a single species at pH 7 (Fig. 5) [39]. These ferric forms have been assigned as species I and II, with species I comprising 45% of the starting ferric DtpAa [39]. The kinetics of Compound I formation reveals species I reacts faster with peroxide than species II (Fig. 5), with species I displaying similar *k*_obs1_ values to those reported at pH 7 [39]. It is important to note that the *k*_obs1_ values for DtpAa are only a few per second, even at peroxide concentrations of 1 mM (Fig. 5) [39], compared with hundreds and tens per second with DtpA and DtpB, respectively [19]. Thus, DtpAa appears only to have limited activity with peroxide. The decay of Compound I to Compound II in DtpAa is independent of peroxide concentration and has a k_obs2_ ~ 0.1 s^−1^ (Fig. 5) [39]. Whilst not reported, the likely source of the electron is from an oxidisable amino acid (e.g. Tyr or Trp) within close proximity to the heme pocket.

### Compound I formation is pH dependent

For both A-type enzymes, Compound I formation has been reported to be pH dependent, with *k*_obs_ values increasing between pH 4 and 7 [39]. An ionisation equilibrium constant (p*K*_a_) of ~ 4.5 has been determined, which has not been attributed to ionisable amino acids, but rather to the p*K*_a_ of the bound peroxide to the Fe^III^-heme [39]. The change in the electrostatic free energy (ΔG_el_) for binding peroxide to a Fe^III^-heme is ~ 34 kJ mol^−1^ which equates to a change in the p*K*_a_ of 6 pH units [39]. Thus, a theoretical decrease in the p*K*_a_ of peroxide from 11.5 to 5.5 would be expected and in line with the experimental p*K*_a_ determined [39]. For DtpB the pH dependency of Compound I formation has yet to be reported.

## DtpA possesses a distal heme pocket that is finely tuned for rapid reactivity with peroxide

Why does DtpA react rapidly and efficiently with peroxide to form Compound I whereas DtpAa shows only limited reactivity despite the apparent structural similarities? To probe further and assist in devising a mechanism of Compound I formation that satisfies the kinetic data and the p*K*_a_ value associated with Compound I formation, studies using site-directed mutagenesis and kinetic isotope experiments have been undertaken.

### Kinetic isotope effect (KIE)

As Compound I formation is associated with the breaking and forming of a O–H bond, then using deuterated peroxide may provide information of whether these reaction steps in DtpA and DtpAa are rate limiting. For DtpA no KIE is observed for Compound I formation [39]. However, for Compound I formation in DtpAa a KIE of ~ 2 was determined, with a similar kinetic profile of *k*_obs1_ versus peroxide concentration as observed in water (Fig. 5) [39]. No KIE was observed for the decay of Compound I to Compound II (*k*_obs2_) in DtpAa. These observations are entirely consistent with the rate determining step for Compound I formation in DtpAa being proton transfer, following the binding of peroxide to the Fe^III^-heme, whereas for DtpA the absence of a KIE indicates proton transfer is faster than peroxide binding [39]. Thus, DtpA has a highly tuned distal site to bind peroxide and facilitate proton transfer to initiate the heterolytic fission of the O–O bond to form Compound I, whereas DtpAa is not optimised [39].

### Effect of the distal heme pocket Asp on Compound I formation

**Fig. 6:**
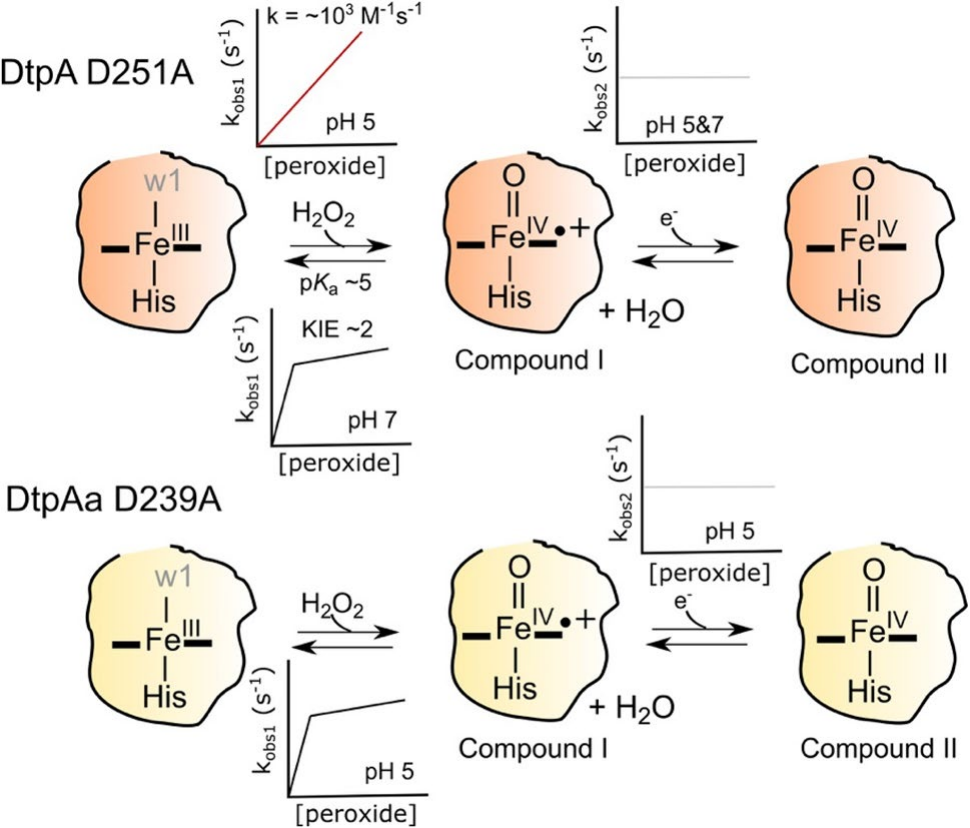
A cartoon summary of the stopped-flow reaction kinetics reported upon reacting the two distal Asp A-type DyP variants with peroxide. For each variant the chemistry that occurs within the heme pocket as determined from absorbance spectrum changes is depicted, along with illustrative plots of *k*_obs_ versus peroxide concentration. The w1 is coloured grey as in the absence of structural data for these variants it is not known whether a water coordinates the Fe^III^-heme. All kinetic experiments were conducted at 25 °C

Substituting the distal Asp residue for an Ala in both DtpA and DtpAa and its effect on Compound I kinetics has been reported [39]. For DtpAa, the kinetic profile on mixing the Asp variant with peroxide was found to be comparable with the wild-type (WT) enzyme, *i.e.* a non-linear dependence on peroxide concentration for Compound I formation and rate limiting behaviour for the decay of Compound I to Compound II (Fig. 6) [39]. However, at pH 5 only one Fe^III^-heme form is now detected (Fig. 6). Notably, the k_obs1_ values remain at a few per second, over a wide range of peroxide concentrations, indicating that whether the distal Asp in DtpAa is present or not, limited peroxide reactivity is retained. This implies that the Asp offers *no* catalytic contribution to Compound I formation.

On the other hand the kinetics of Compound I formation with the DtpA distal Asp variant differ to that of the WT enzyme [39]. Now a two-state transition that passes through Compound I to a Compound II species, similar to that observed spectroscopically for DtpAa is observed (Fig. 6) [39]. In accordance with WT DtpAa and its Asp variant, the *k*_obs1_ values are now only a few per second compared to hundreds per second for WT DtpA [39]. At pH 5 a linear dependence of k_obs1_ with increasing peroxide concentrations is reported, yielding a *k*_cmpdI_ of 4 × 10^3^ M^−1^ s^−1^ (Fig. 6). Such a value is analogous to those reported for oxidation of water-soluble Fe^III^-tetraphenyl-porphyrins with peroxide [70], and strongly suggests that unlike in DtpAa, the distal Asp in DtpA has a catalytic role. Furthermore, the linear dependence of *k*_obs_ with increasing peroxide concentration indicates that, in this variant, the rate determining step is still peroxide binding to the Fe^III^-heme, with proton transfer faster than peroxide binding [39]. However, at pH 7 the k_obs1_ values become rate limited at high peroxide concentration and a KIE of ~ 2 is observed, now indicating that proton transfer is rate limiting (Fig. 6). Thus, removal of the distal Asp in DtpA disturbs a fine balance between whether peroxide binding or proton transfer are rate limiting Compound I formation [39]. Finally, Compound I formation for the distal Asp variants in both A-type enzymes is also pH dependent, with a p*K*_a_ of ~ 5.5 determined [39]. This value is slightly elevated compared to the WT enzymes but is in line with expectations considering removal of a nearby negative charge.

### Mechanism of Compound I formation in DtpA

**Fig. 7:**
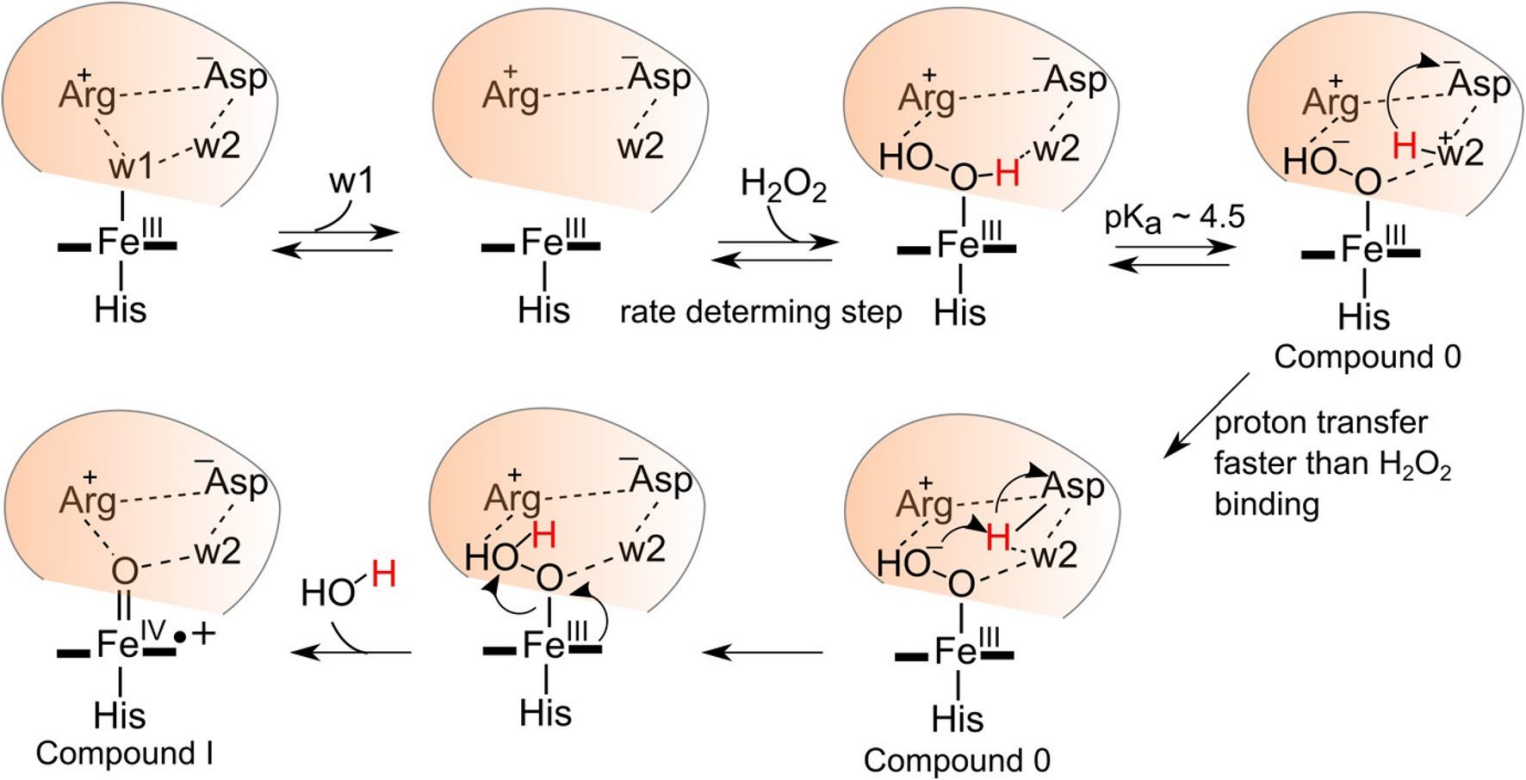
The catalytic mechanism of Compound I formation in DtpA based on structural and kinetic data. The H^α^ proton of the peroxide is shown in red and H-w2^+^ represents a hydronium ion. For a complete description of the individual steps see main text

The kinetic studies strongly advocate DtpA as having a finely tuned distal site to activate peroxide and rapidly form Compound I, whereas DtpAa appears to form Compound I in a non-facilitated manner [39]. A catalytic mechanism for DtpA that accounts for the reported structural and kinetics studies has been proposed and is depicted in Fig. 7. The distal Asp is assigned in an anionic state, complying with experimental evidence from other DyPs to suggest a p*K*_a_ <  < 4 [37, 71]. The first step in the mechanism is accounted for by the validated Fe^III^-heme resting state X-ray structure (Fig. 3). However, w1 must be highly labile as inferred from the linear dependence of *k*_obs_ for Compound I formation, which in the absence of a KIE indicates that peroxide binding is the rate determining step in the overall mechanism. To account for the experimentally observed p*K*_a_ in the mechanism, deprotonation of the peroxide to form a hydronium ion and Compound 0 is suggested (Fig. 7) [39]. Here, w2 as identified in the Fe^III^-heme X-ray structure H-bonds to the distal Asp (Fig. 3) and can act as a convenient conduit to move a proton, by first forming a hydronium ion and then transiently moving the proton onto the Asp for transfer to the − OH^β^ prior to heterolysis of the of the O–O bond (Fig. 7). It is noted that in the DtpA Asp variant a comparable p*K*_a_ is observed and this is also attributed to the formation of a hydronium ion [39]. However, in the absence of the Asp this hydronium ion is not optimally configured for proton-transfer, but nevertheless at pH 5 proton-transfer remains faster than peroxide binding [39]. Equilibrium molecular dynamics and free energy calculations in HRP have demonstrated that a water-His unit is energetically favoured to move a proton onto the distal OH of the bound peroxide rather than direct proton movement involving only the distal His [28]. Thus, a similar scenario may be envisaged with DtpA. The remaining proton and electron transfer steps are rapid (no KIE) and result in Compound I formation (Fig. 7).

Based on the mechanism depicted in Fig. 7, a strong case can be made for Compound I formation in DtpA to be driven by the stereochemistry of the w2-Asp unit. Despite DtpAa having the w2-Asp unit (Fig. 3), it is proposed that the positional shift identified in the validated X-ray structure perturbs the optimal stereochemistry required for a catalytic role [28]. Based on the interpretation of the kinetic studies for DtpAa it can be argued that the positional change of the w2-Asp unit introduces a steric constraint into the distal pocket that hinders the rate of peroxide binding, destabilises the Fe^III^–OH–OH complex and perturbs proton-transfer [28]. Thus, DtpA is a highly reactive peroxidase, whereas DtpAa, because of the subtle positional change of the w2-Asp unit, is not. This explanation highlights that ensuring the X-ray structures are determined free of site-specific radiation damage, then insights into mechanism can be achieved and advanced with confidence.

The observation of this major difference in reactivity from otherwise highly similar enzymes with active sites that appear at first glance essentially identical, highlights the need for kinetic characterisation of peroxidase activity and suggests caution in assigning peroxidase activity to DyPs purely based on sequence homology or homology modelling approaches.

## DtpB Compound I structure

The chemical nature of the ferryl species assigned as Compounds I and II in peroxidases has attracted much discussion as to whether they are formulated as an unprotonated Fe^IV^ = O or a protonated Fe^IV^–OH species [18]. The reactivity of these ferryl species is controlled by protonation. There is a long experimental history associated with efforts to assign the chemical nature of these ferryl species using spectroscopy (e.g. EXAFS (Extended X-ray Absorption Fine Structure), resonance Raman and Mössbauer), X-ray crystallography and more recently neutron crystallography, which not only eliminates radiation damage, but also has the advantage of being able to observe nuclear density attributed to proton positions. A detailed history of the experimental efforts to define the nature of ferryl heme has been reviewed by Raven and Moody [18]. Regardless of the method used, the challenge remains to isolate peroxidases exclusively in either the ‘pure’ Compound I or Compound II states [72]. These states are often unstable, as evident with DtpA and particularly for DtpAa, with mixtures of states in part causing erroneous interpretation as to the nature of the ferryl species. DtpB on the other hand has provided an opportunity to obtain an intact structure of a “green” Compound I species (*i.e.* contains a porphyrin π-cation radical) owing to the long Compound I life-time.

**Fig. 8:**
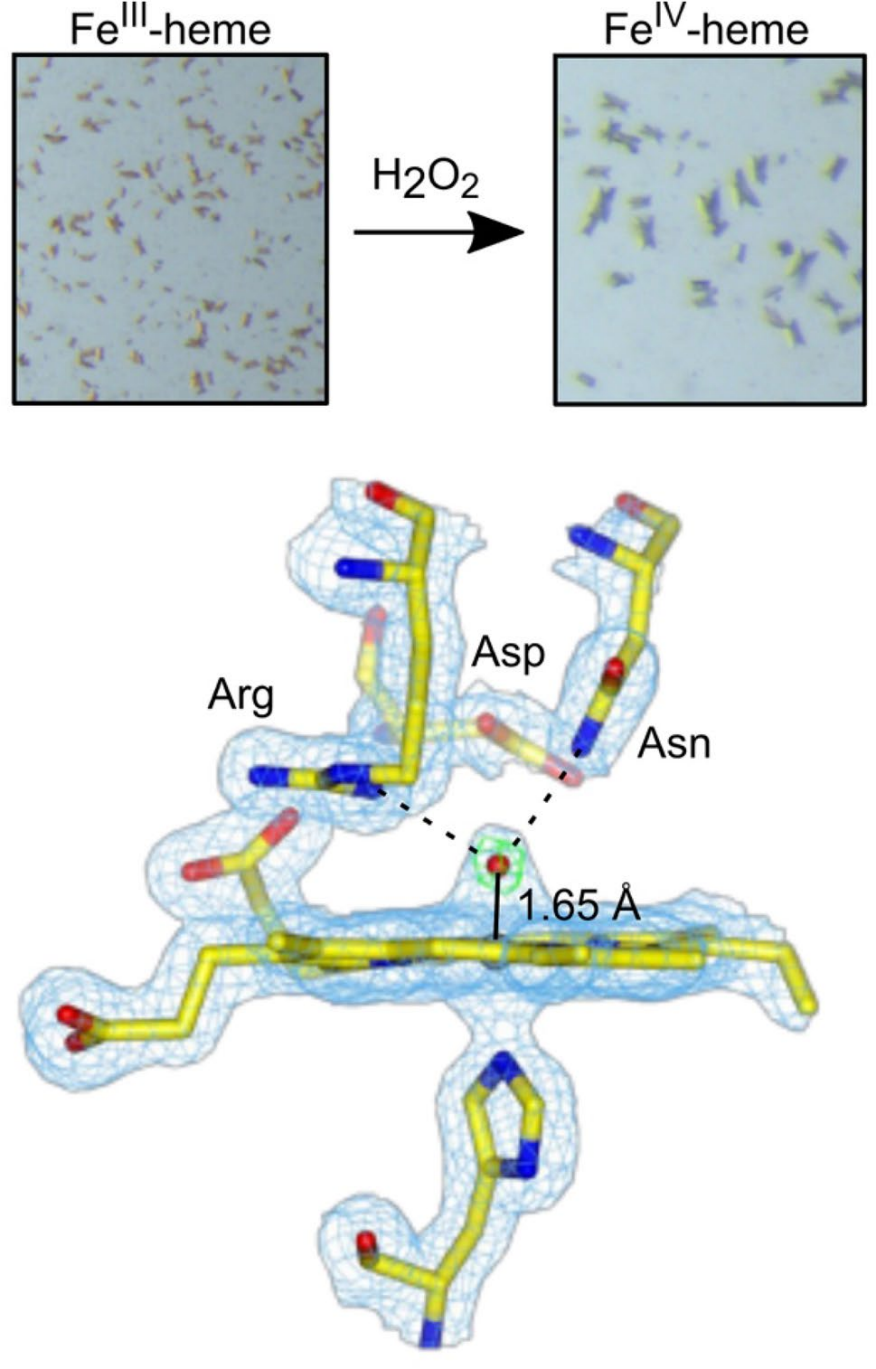
Top panel: Microcrystals (~ 10 μm) of DtpB in the Fe^III^-heme state (brown) and in the Fe^IV^-heme state (green) following addition of H_2_O_2_. Bottom panel: Heme site of DtpB (chain A) determined by SFX at room temperature following addition of H_2_O_2_ to the Fe^III^-heme microcrystals [35]. The *2F*_*o*_*-F*_*c*_ electron density map (blue) contoured at 1.4 σ and the *F*_*o*_*-F*_*c*_ omit map (green) contoured at ± 10 σ, calculated after refinement, omitting the oxygen atom (red sphere). H-bond interactions involving the Fe^IV^ = O are indicated in dashed lines. PDB code used 6YRD

Green crystals of DtpB could be generated upon addition of peroxide to ferric crystals (Fig. 8). Both the fixed-target room temperature SFX approach (zero dose) and a 100-K composite multi-crystal strategy with in situ monitoring using microspectrophotometry were used to determine the structure of this species (Table 2). As noted previously with the Fe^III^-heme structures, the stereochemistry in the distal pocket on comparing the structures from the two approaches used were identical within experimental error [35]. An electron density feature was observed on the distal side of the heme, into which an oxygen atom was readily modelled (Fig. 8). Assigning the ferryl species as protonated or unprotonated can be implied indirectly by the Fe–O bond length, which is often used to determine bond order, *i.e.* single or double bond and thus protonation state. For one of the monomers in the asymmetric crystallographic unit (total of six) a Fe^IV^–O bond length of 1.65 Å is reported, in both the SFX and 100 K composite structures [35]. This bond length falls within the expected range for a Fe^IV^ = O species and is line with the most reliable bond lengths (*i.e.* X-ray structures agree with spectroscopy) determined for Compound I species in proteins (1.63 to 1.73 Å) [52–54, 56, 73–75]. The formation of Compound I in DtpB coincides with the formation of a new H-bond network centred around the oxo group which accepts H-bonds from the amino group of Asn245 and the N^η1^ atom of Arg243 (Fig. 8).

In the other five monomers of the crystallographic asymmetric unit of DtpB, variation in the Fe–O bond length (1.70–1.89 Å) at both room temperature and 100 K is reported [35]. Lengthening of the Fe^IV^–O bond can indicate the formation of an Fe^IV^–OH or Fe^III^–OH species [76, 77]. For an Fe^IV^–OH species an Fe–O bond distance determined through spectroscopic approaches lies between 1.76 and 1.82 Å [78, 79], whereas Fe^III^–OH is longer > 1.85 Å [77]. In peroxidases and globins with histidine-ligated heme, no spectroscopic evidence for a Fe^IV^–OH Compound II species has been reported, in accord with the chemistry of peroxidases (*i.e.* one-electron oxidation of substrates as opposed to C–H bond activations as is the case in cytochrome P450s). However, the observation of positive nuclear density next to the Fe–O bond in the neutron structure of APX Compound II supports a hydroxide species [80]. A Fe–O bond distance of 1.88 Å has been determined and the investigators assign this species as a Fe^IV^–OH [80]. More recently a XFEL study (100 K) with APX Compound II reveals a Fe–O bond distance of 1.87 Å [72] and thus confirming the distance obtained by neutron crystallography [80]. These zero dose structural studies suggest that the Compound II species generated in APX could be an outlier amongst peroxidases, in that an Fe^IV^–OH species is formed. However, this view contrasts to spectroscopy data obtained with EXAFS and Mössbauer [81], and more recently NRVS (Nuclear Resonance Vibrational Spectroscopy) [82] where an Fe–O bond distance of 1.68 Å for APX Compound II is determined, consistent with an unprotonated Fe^IV^ = O species [77]. Thus, a disagreement between spectroscopy data and zero dose structures exists. For DtpB the heterogeneity in the Fe–O bond lengths present in the other asymmetric unit monomers, is at present difficult to explain. X-ray induced reduction would appear unlikely considering the low absorbed dose for the 100 K structure (Table 2) and that the SFX approach safeguards against site-specific radiation damage occurring. Furthermore, the in situ microspectrophotometry measurements of the crystal after exposure reveal no evidence for a mixed valence species [35]. However, the increase in bond lengths would suggest a partial onset of reduction of the Fe^IV^ = O species. It may be that endogenous reductants that depend on local environment which differ in each molecule of the asymmetric unit could be causing these changes. Further investigation through the acquisition of dose series data (*i.e.* by feeding electrons into the system via radiolysis) could shed light into the heterogeneity of Fe–O bond lengths observed amongst the monomers in the DtpB crystallographic asymmetric unit.

## The distal Arg facilitates O–O heterolysis in DtpB

The ‘dry’ distal heme site identified in Fe^III^-heme DtpB reveals that the water-mediated Asp mechanism utilised for Compound I formation in DtpA cannot occur in this B-type DyP [39]. However, Compound I in DtpB does form rapidly. No KIE is observed (Lučić unpublished data) and thus peroxide binding is the rate determining step with proton transfer being faster than the binding of peroxide. Furthermore, the *k*_cmpdI_ is significantly higher than for a non-facilitated Compound I formation as observed for DtpAa and Fe^III^-tetraphenyl-porphyrins, so even in the absence of a water-Asp unit a catalytic role for either the Asp or Arg would be expected.

Substitution of the distal Asp and Arg residues with an Ala has helped to elucidate their roles in Compound I formation in DtpB [35]. For the Asp variant, the kinetic and spectral properties of Compound I formation are identical to WT DtpB. However, in the Arg variant, Compound I forms (over many minutes) and decays back to the Fe^III^-heme species. Thus, removal of the distal Arg slows Compound I formation and renders it unstable [35]. These data have been taken to indicate that in DtpB Arg is selected to facilitate Compound I formation. Interestingly, the presence of a distal pocket Asn is noted in two other DyPs for which reports suggest the formation of Compound I is Arg dependent [33, 83]. Thus, based on the structural and kinetic studies with DtpB, a combination of a ‘dry’ site, and the presence of an Asn in the distal pocket could be part of the structural criteria that selects Arg for proton movement on peroxide binding to the Fe^III^-heme [35].

**Fig. 9:**
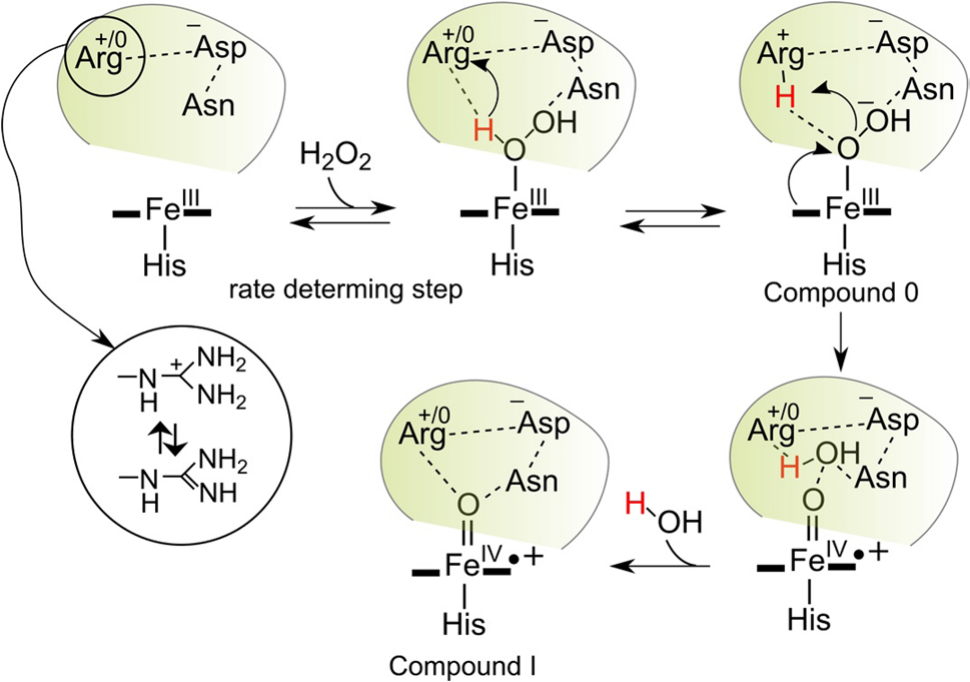
The catalytic mechanism of Compound I formation in DtpB based on structural and kinetic data. See main text for a discussion of the individual steps

Can an Arg facilitate general acid/base chemistry? Examples of Arg acting as a catalytic base have been reported in enzymes including metalloenzymes [84], despite the guanidinium group having a p*K*_a_ of 13.8 [85], and thus protonated at neutral pH. Furthermore, several neutron structures have provided evidence for the existence of guanidine (neutral) side chains [86–88], including APX when bound to its substrate ascorbic acid [89]. A recent report has highlighted the need for an Arg to boost peroxidase activity in artificial metalloenzyme containing a manganese porphyrin that possesses dye-decolorizing activity [90]. An excellent review by Schlippe and Hedstrom [91], surveys enzymes which by implication use Arg as an acid/base catalyst. A key factor to consider is what fraction of an Arg must be deprotonated for effective general base catalysis to occur. Providing this fraction is kinetically competent it will be sufficient to drive catalysis. Two further considerations are of note. The first, is that Arg residues that have been identified as potential bases are H-bonded to carboxylate groups and second, they can link, through H bonding networks to the bulk solvent [86–88, 91]. The distal Arg in DtpB satisfies both these criteria; it is H-bonded to the carboxylates of the heme propionate-7 group and its N^ε^ atom is H-bonded to a water which is part of the H-bonded water network that leads to the surface opening at the γ face of the heme, as identified using CAVER (Fig. 4). It has been suggested that the carboxylates of the Arg-carboxylate motif catalyses proton exchange, which is rapid and efficient owing to the communication with bulk solvent [86–88, 91]. This would ensure that the active deprotonated non-planar (neutral) form is in rapid equilibrium with the inactive planar (charged) form, so at any one time a fraction of the active enzyme is present. Taking these factors into account along with the structural and kinetic data a mechanism for Compound I formation for DtpB can be proposed (Fig. 9).

## Consequences of a ‘dry’ distal pocket

The ‘dry’ distal heme pocket identified in Fe^III^-heme DtpB remains in the Compound I structure. This indicates that the water molecule coproduced on Compound I formation is released from the distal pocket, emphasising the importance of the ease of egress allowed by solvent exposure of the heme site. It has been proposed that the release or retention of the coproduced water molecule upon Compound I formation may influence whether a two-electron reduction of Compound I occurs (a ‘dry’ site; water released) such as in catalases or two one-electron equivalent processes (a ‘wet site; water retained) resulting first in the Compound II intermediate and then the ferric resting state, as in peroxidases [92]. This concept of a redox pathway switching mechanism arose in the early 2000’s when more X-ray structures of peroxidases and catalases were becoming available. However, as commented on above, site-specific radiation damage is now known to be a confounding factor in these earlier structures. Thus, despite initial insights into the presence or absence of heme distal pocket water molecules that underpins this redox switching theory, an air of caution should remain as in DyPs, distal pocket waters can move and disappear upon a change of redox state caused by radiation damage [45, 49]. Nevertheless, the ‘dry’ versus ‘wet’ theory is compelling, but at the same time has yet to be unequivocally proven. Structural evidence to support and test the theory has been lacking, but the DtpB Compound I structure provides a starting point to test whether a ‘dry’ site does indeed lead to simultaneous two-electron reduction to the ferric state.

Finally, a ‘dry’ distal site in DtpB may have consequences for reactivity. First it has been recognised that the polarity of the distal heme pocket promotes charge separation, as is required for heterolytic cleavage of the O–O bond, whereas nonpolar environments promote homolytic cleavage. In myoglobin, ~ 30% of peroxide bond-breaking occurs via homolytic cleavage [93]. The absence of water in the distal pocket in DtpB will certainly affect the distal pocket polarity. However, on reaction with peroxide an electronic absorption spectrum consistent with a Compound I species forms as expected for heterolytic cleavage, with no detection of a Compound II spectrum [35], which would be expected if homolytic cleavage had occurred. Once Compound I is formed in DtpB, it is highly stable, and thus unreactive. It is known that preparing Compound I-like derivatives in ferric porphyrin complexes in non-aqueous solvents gives rise to very stable ferryl species in the absence of an electron donor [94]. However, by increasing the polarity through addition of water, the ferryl species are markedly destabilised suggesting that a factor for Compound I stability not only in these DyPs but also peroxidases in general may be linked to whether after forming Compound I the distal pocket is ‘wet’ or ‘dry’.

## Future directions

### Time-resolved crystallography

The combination of determining redox state validated X-ray structures coupled with stopped-flow kinetics and site-directed mutagenesis studies has proven key to understanding the mechanistic subtleties of Compound I formation in this set of DyPs. However, only the structures and mechanisms associated with the formation of the first ferryl intermediate species, Compound I, have to date been reported. No structural studies on a DyP Compound II species are available and approaches to address this would be a next step. It is noted that cryo-trapping of ‘pure’ Compound II in APX and CcP has been possible [72, 80], and such an approach may be amenable in this set of DyPs. However, whilst obtaining ‘static’ structures of ferryl species are clearly not without challenges, a grand challenge would be to capture the intact structures of the peroxidatic cycle in ‘real-time’. In this respect the on-going developments in room temperature time resolved X-ray crystallography open an exciting area to exploit the visualisation of structural intermediates in reaction cycles [95, 96]. The initial knowledge of the solution state kinetics for this set of DyPs form the basis for planning time-resolved crystallography experiments. For example, on mixing peroxide with DtpAa, Compound I and Compound II species are sequentially formed (Fig. 5), with their life-times amenable to time-resolved crystallography approaches. It is, therefore, conceivable that mixing peroxide with DtpAa microcrystals will allow time-resolved structural characterisation of the peroxidatic pathway. Both low-dose synchrotron and XFEL experiments can achieve this, with a key requirement being spectroscopic validation of a particular intermediate state occurring at a particular time point, which may well be different to the reaction time point observed in stopped-flow solution kinetics. However, recent methodological developments in this area such as the use of X-ray emission spectroscopy (XES) arising from the same X-ray pulse as used for diffraction data collection, will allow for the validation of structures corresponding to particular time points for the reaction in the crystal to be determined. Mixing approaches recently developed include microfluidic [97, 98] or larger scale turbulent mixing [99] as well as drop-on-drop mixing either in fixed targets [100] or on a moving tape [101]. Taking advantage of these methods would also give scope for DtpA and DtpB to be studied by first generating Compound I in situ followed by injection into the system of an electron donor or by controlled use of the X-ray beam.

### Involvement of protons

The movement of electrons in metalloenzymes is often accompanied by movement of protons. As indicated several times in this review, proton movement in the peroxidase cycle is important and centrally involved in mechanism. Whilst a major focus has been to ensure that redox states of crystal structures are validated, questions as to the protonation state of key residues around the distal heme pocket at different stages of the catalytic cycle remain. Protonation may be directly observed by neutron crystallography for stable or cryo-trapped intermediates, although neutron diffraction experiments remain challenging [102]. However, neutron experiments have been successfully used to probe the protonation state of cryo-trapped ferryl states in CcP and APX [74, 80], as well as their distal pocket residues, and more recently to visualise the proton transfer pathways in APX bound to its substrate ascorbate [89]. Very recently, atomic resolution XFEL structures from large crystals at 100 K have been produced of CcP using serial femtosecond rotation crystallography (SF-ROX) with the ultrahigh resolution allowing protonation states to be assessed [72]. Application of this approach to DyPs could be very informative, particularly as the *S. lividans* DyP macro-crystals will diffract to < 1.2 Å using a synchrotron X-ray source.

## References

[1] Kim SJ, Shoda M (1999). Appl Environ Microbiol.

[2] Singh R, Eltis LD (2015). Arch Biochem Biophys.

[3] Sugano Y, Muramatsu R, Ichiyanagi A, Sato T, Shoda M (2007). J Biol Chem.

[4] Hofbauer S, Pfanzagl V, Michlits H, Schmidt D, Obinger C, Furtmüller PG (2021) Biochim Biophys Acta Proteins Proteom 1869:140536 10.1016/j.bbapap.2020.140536 PMC7611857 32891739

[5] Ogola HJ, Kamiike T, Hashimoto N, Ashida H, Ishikawa T, Shibata H, Sawa Y (2009). Appl Environ Microbiol.

[6] Yoshida T, Sugano Y (2015). Arch Biochem Biophys.

[7] Liers C, Bobeth C, Pecyna M, Ullrich R, Hofrichter M (2010). Appl Microbiol Biotechnol.

[8] Liers C, Pecyna MJ, Kellner H, Worrich A, Zorn H, Steffen KT, Hofrichter M, Ullrich R (2013). Appl Microbiol Biotechnol.

[9] Sugawara K, Igeta E, Amano Y, Hyuga M, Sugano Y (2019). AMB Express.

[10] Sutter M, Boehringer D, Gutmann S, Günther S, Prangishvili D, Loessner MJ, Stetter KO, Weber-Ban E, Ban N (2008). Nat Struct Mol Biol.

[11] Rahmanpour R, Bugg TD (2013). FEBS J.

[12] Contreras H, Joens MS, McMath LM, Le VP, Tullius MV, Kimmey JM, Bionghi N, Horwitz MA, Fitzpatrick JA, Goulding CW (2014). J Biol Chem.

[13] Giessen TW (2016). Curr Opin Chem Biol.

[14] Giessen TW, Silver PA (2017). Nat Microbiol.

[15] Tang Y, Mu A, Zhang Y, Zhou S, Wang W, Lai Y, Zhou X, Liu F, Yang X, Gong H, Wang Q, Rao Z (2021) Proc Nat Acad Sci USA 118:e2025658118 10.1073/pnas.2025658118 PMC8072242 33853951

[16] Groves JT (2006). J Inorg Biochem.

[17] Winkler JR, Gray HB (2011). Electronic structures of oxo-metal ions.

[18] Moody PCE, Raven EL (2018). Acc Chem Res.

[19] Chaplin AK, Wilson MT, Worrall JAR (2017) Dalton Trans 46:9420–9429 10.1039/c7dt01144j 28695933

[20] Theorell H (1941). Enzymologia.

[21] Keilin D, Hartree EF (1951). Biochem J.

[22] Dunford HB (2010). Peroxidases and catalases: Biochemistry, biophysics, biotechnology, and physiology.

[23] Poulos TL (2014). Chem Rev.

[24] Poulos TL, Kraut J (1980). J Biol Chem.

[25] Erman JE, Vitello LB, Miller MA, Shaw A, Brown KA, Kraut J (1993). Biochemistry.

[26] Howes BD, Rodriguez-Lopez JN, Smith AT, Smulevich G (1997). Biochemistry.

[27] Rodriguez-Lopez JN, Lowe DJ, Hernandez-Ruiz J, Hiner AN, Garcia-Canovas F, Thorneley RN (2001). J Am Chem Soc.

[28] Vidossich P, Fiorin G, Alfonso-Prieto M, Derat E, Shaik S, Rovira C (2010). J Phys Chem B.

[29] Baek HK, Van Wart HE (1989). Biochemistry.

[30] Svistunenko DA, Reeder BJ, Wankasi MM, Silaghi-Dumitrescu RL, Cooper CE, Rinaldo S, Cutruzzola F, Wilson MT (2007) Dalton Trans 28:840–850 10.1039/b615770j 17297511

[31] Sugano Y (2009). Cell Mol Life Sci.

[32] Hiner AN, Raven EL, Thorneley RN, Garcia-Canovas F, Rodriguez-Lopez JN (2002). J Inorg Biochem.

[33] Singh R, Grigg JC, Armstrong Z, Murphy ME, Eltis LD (2012). J Biol Chem.

[34] Mendes S, Brissos V, Gabriel A, Catarino T, Turner DL, Todorovic S, Martins LO (2015). Arch Biochem Biophys.

[35] Lučić M, Svistunenko DA, Wilson MT, Chaplin AK, Davy B, Ebrahim A, Axford D, Tosha T, Sugimoto H, Owada S, Dworkowski FSN, Tews I, Owen RL, Hough MA, Worrall JAR (2020). Angew Chem Int Ed Engl.

[36] Chen C, Shrestha R, Jia K, Gao PF, Geisbrecht BV, Bossmann SH, Shi J, Li P (2015). J Biol Chem.

[37] Shrestha R, Huang GC, Meekins DA, Geisbrecht BV, Li P (2017). ACS Catal.

[38] Pfanzagl V, Bellei M, Hofbauer S, Laurent C, Furtmuller PG, Oostenbrink C, Battistuzzi G, Obinger C (2019) J Inorg Biochem 199:110761 10.1016/j.jinorgbio.2019.110761 31325671

[39] Lucic M, Chaplin AK, Moreno-Chicano T, Dworkowski FSN, Wilson MT, Svistunenko DA, Hough MA, Worrall JAR (2020) Dalton Trans 49:1620–1636 10.1039/c9dt04583j 31942590

[40] Petrus ML, Vijgenboom E, Chaplin AK, Worrall JA, van Wezel GP, Claessen D (2016) Open Biol 6:150149 10.1098/rsob.150149 PMC4736821 26740586

[41] Ahmad M, Roberts JN, Hardiman EM, Singh R, Eltis LD, Bugg TD (2011). Biochemistry.

[42] Liu X, Du Q, Wang Z, Zhu D, Huang Y, Li N, Wei T, Xu S, Gu L (2011). J Biol Chem.

[43] Roberts JN, Singh R, Grigg JC, Murphy ME, Bugg TD, Eltis LD (2011). Biochemistry.

[44] Liu X, Yuan Z, Wang J, Cui Y, Liu S, Ma Y, Gu L, Xu S (2017). Biochem Biophys Res Commun.

[45] Pfanzagl V, Beale JH, Michlits H, Schmidt D, Gabler T, Obinger C, Djinović-Carugo K, Hofbauer S (2020). J Biol Chem.

[46] Burmeister WP (2000). Acta crystallographica Section D. Biol Crystallography.

[47] Ravelli RB, McSweeney SM (2000). Structure.

[48] Weik M, Ravelli RB, Kryger G, McSweeney S, Raves ML, Harel M, Gros P, Silman I, Kroon J, Sussman JL (2000). Proc Natl Acad Sci USA.

[49] Kekilli D, Moreno-Chicano T, Chaplin AK, Horrell S, Dworkowski FSN, Worrall JAR, Strange RW, Hough MA (2017). IUCrJ.

[50] Henderson R (1995). Q Rev Biophys.

[51] Owen RL, Rudiño-Piñera E, Garman EF (2006). Proc Natl Acad Sci USA.

[52] Berglund GI, Carlsson GH, Smith AT, Szoke H, Henriksen A, Hajdu J (2002). Nature.

[53] Meharenna YT, Doukov T, Li H, Soltis SM, Poulos TL (2010). Biochemistry.

[54] Gumiero A, Metcalfe CL, Pearson AR, Raven EL, Moody PC (2011). J Biol Chem.

[55] Chaplin AK, Chicano TM, Hampshire BV, Wilson MT, Hough MA, Svistunenko DA, Worrall JAR (2019) Chemistry 25:6141–6153 10.1002/chem.201806290 30945782

[56] Chreifi G, Baxter EL, Doukov T, Cohen AE, McPhillips SE, Song J, Meharenna YT, Soltis SM, Poulos TL (2016). Proc Natl Acad Sci USA.

[57] Ebrahim A, Moreno-Chicano T, Appleby MV, Chaplin AK, Beale JH, Sherrell DA, Duyvesteyn HME, Owada S, Tono K, Sugimoto H, Strange RW, Worrall JAR, Axford D, Owen RL, Hough MA (2019). IUCrJ.

[58] Shrestha R, Jia K, Khadka S, Eltis LD, Li P (2021). ACS Catal.

[59] Neutze R, Wouts R, van der Spoel D, Weckert E, Hajdu J (2000). Nature.

[60] Chapman HN, Fromme P, Barty A, White TA, Kirian RA, Aquila A, Hunter MS, Schulz J, DePonte DP, Weierstall U, Doak RB, Maia FR, Martin AV, Schlichting I, Lomb L, Coppola N, Shoeman RL, Epp SW, Hartmann R, Rolles D, Rudenko A, Foucar L, Kimmel N, Weidenspointner G, Holl P, Liang M, Barthelmess M, Caleman C, Boutet S, Bogan MJ, Krzywinski J, Bostedt C, Bajt S, Gumprecht L, Rudek B, Erk B, Schmidt C, Hömke A, Reich C, Pietschner D, Strüder L, Hauser G, Gorke H, Ullrich J, Herrmann S, Schaller G, Schopper F, Soltau H, Kühnel KU, Messerschmidt M, Bozek JD, Hau-Riege SP, Frank M, Hampton CY, Sierra RG, Starodub D, Williams GJ, Hajdu J, Timneanu N, Seibert MM, Andreasson J, Rocker A, Jönsson O, Svenda M, Stern S, Nass K, Andritschke R, Schröter CD, Krasniqi F, Bott M, Schmidt KE, Wang X, Grotjohann I, Holton JM, Barends TR, Neutze R, Marchesini S, Fromme R, Schorb S, Rupp D, Adolph M, Gorkhover T, Andersson I, Hirsemann H, Potdevin G, Graafsma H, Nilsson B, Spence JC (2011). Nature.

[61] Chapman HN, Caleman C, Timneanu N (2014). Philos Trans R Soc Lond B Biol Sci.

[62] Spence JC (2014). Faraday Discuss.

[63] Schlichting I (2015). IUCrJ.

[64] Moreno-Chicano T, Ebrahim A, Axford D, Appleby MV, Beale JH, Chaplin AK, Duyvesteyn HME, Ghiladi RA, Owada S, Sherrell DA, Strange RW, Sugimoto H, Tono K, Worrall JAR, Owen RL, Hough MA (2019). IUCrJ.

[65] Fraser JS, van den Bedem H, Samelson AJ, Lang PT, Holton JM, Echols N, Alber T (2011). Proc Natl Acad Sci USA.

[66] Potterton L, Agirre J, Ballard C, Cowtan K, Dodson E, Evans PR, Jenkins HT, Keegan R, Krissinel E, Stevenson K, Lebedev A, McNicholas SJ, Nicholls RA, Noble M, Pannu NS, Roth C, Sheldrick G, Skubak P, Turkenburg J, Uski V, von Delft F, Waterman D, Wilson K, Winn M, Wojdyr M (2018). Acta crystallographica Section D. Structural biology.

[67] Chovancova E, Pavelka A, Benes P, Strnad O, Brezovsky J, Kozlikova B, Gora A, Sustr V, Klvana M, Medek P, Biedermannova L, Sochor J, Damborsky J (2012) PLoS Comput Biol 8:e1002708 10.1371/journal.pcbi.1002708 PMC3475669 23093919

[68] Holzwarth JF, Meyer F, Pickard M, Dunford HB (1988). Biochemistry.

[69] Rodriguez-Lopez JN, Smith AT, Thorneley RN (1996). J Biol Chem.

[70] Bruice TC (1991). Acc Chem Res.

[71] Pfanzagl V, Nys K, Bellei M, Michlits H, Mlynek G, Battistuzzi G, Djinovic-Carugo K, Van Doorslaer S, Furtmuller PG, Hofbauer S, Obinger C (2018). J Biol Chem.

[72] Kwon H, Basran J, Pathak C, Hussain M, Freeman SL, Fielding AJ, Bailey AJ, Stefanou N, Sparkes HA, Tosha T, Yamashita K, Hirata K, Murakami H, Ueno G, Ago H, Tono K, Yamamoto M, Sawai H, Shiro Y, Sugimoto H, Raven E, Moody PCE (2021). Angew Chem Int Ed Engl.

[73] Stone KL, Behan RK, Green MT (2005). Proc Natl Acad Sci USA.

[74] Casadei CM, Gumiero A, Metcalfe CL, Murphy EJ, Basran J, Concilio MG, Teixeira SC, Schrader TE, Fielding AJ, Ostermann A, Blakeley MP, Raven EL, Moody PC (2014) Science 345:193–197 10.1126/science.1254398 25013070

[75] Yosca TH, Langston MC, Krest CM, Onderko EL, Grove TL, Livada J, Green MT (2016). J Am Chem Soc.

[76] Behan RK, Green MT (2006). J Inorg Biochem.

[77] Green MT (2006). J Am Chem Soc.

[78] Stone KL, Behan RK, Green MT (2006). Proc Natl Acad Sci USA.

[79] Newcomb M, Halgrimson JA, Horner JH, Wasinger EC, Chen LX, Sligar SG (2008). Proc Natl Acad Sci USA.

[80] Kwon H, Basran J, Casadei CM, Fielding AJ, Schrader TE, Ostermann A, Devos JM, Aller P, Blakeley MP, Moody PC, Raven EL (2016). Nat Commun.

[81] Ledray AP, Krest CM, Yosca TH, Mittra K, Green MT (2020). J Am Chem Soc.

[82] Ledray AP, Mittra K, Green MT (2021) J Inorg Biochem (**in press**) 10.1016/j.jinorgbio.2021.111548 PMC11890179 34481347

[83] Mendes S, Catarino T, Silveira C, Todorovic S, Martins LO (2015). Catal Sci Technol.

[84] Evans RM, Brooke EJ, Wehlin SA, Nomerotskaia E, Sargent F, Carr SB, Phillips SE, Armstrong FA (2016). Nat Chem Biol.

[85] Fitch CA, Platzer G, Okon M, Garcia-Moreno BE, McIntosh LP (2015). Protein Sci.

[86] Yamaguchi S, Kamikubo H, Kurihara K, Kuroki R, Niimura N, Shimizu N, Yamazaki Y, Kataoka M (2009). Proc Natl Acad Sci USA.

[87] Hiromoto T, Meilleur F, Shimizu R, Shibazaki C, Adachi M, Tamada T, Kuroki R (2017). Protein Sci.

[88] Yonezawa K, Shimizu N, Kurihara K, Yamazaki Y, Kamikubo H, Kataoka M (2017). Sci Rep.

[89] Kwon H, Basran J, Devos JM, Suardiaz R, van der Kamp MW, Mulholland AJ, Schrader TE, Ostermann A, Blakeley MP, Moody PCE, Raven EL (2020). Proc Natl Acad Sci USA.

[90] Markel U, Sauer DF, Wittwer M, Schiffels J, Cui H, Davari MD, Kröckert KW, Herres-Pawlis S, Okuda J, Schwaneberg U (2021). ACS Catal.

[91] Guillen Schlippe YV, Hedstrom L (2005) Arch Biochem Biophys 433:266–278 10.1016/j.abb.2004.09.018 15581582

[92] Jones P (2001). J Biol Chem.

[93] Allentoff AJ, Bolton JL, Wilks A, Thompson JA, Demontellano PRO (1992). J Am Chem Soc.

[94] Felton RH, Owen GS, Dolphin D, Forman A, Borg DC, Fajer J (1973). AnnNY AcadSci.

[95] Orville AM (2020). Curr Opin Struct Biol.

[96] Pearson AR, Mehrabi P (2020). Curr Opin Struct Biol.

[97] Calvey GD, Katz AM, Zielinski KA, Dzikovski B, Pollack L (2020). Anal Chem.

[98] Knoška J, Adriano L, Awel S, Beyerlein KR, Yefanov O, Oberthuer D, Peña Murillo GE, Roth N, Sarrou I, Villanueva-Perez P, Wiedorn MO, Wilde F, Bajt S, Chapman HN, Heymann M (2020) Nat Commun 11:657 10.1038/s41467-020-14434-6 PMC6994545 32005876

[99] Olmos JL, Pandey S, Martin-Garcia JM, Calvey G, Katz A, Knoska J, Kupitz C, Hunter MS, Liang M, Oberthuer D, Yefanov O, Wiedorn M, Heyman M, Holl M, Pande K, Barty A, Miller MD, Stern S, Roy-Chowdhury S, Coe J, Nagaratnam N, Zook J, Verburgt J, Norwood T, Poudyal I, Xu D, Koglin J, Seaberg MH, Zhao Y, Bajt S, Grant T, Mariani V, Nelson G, Subramanian G, Bae E, Fromme R, Fung R, Schwander P, Frank M, White TA, Weierstall U, Zatsepin N, Spence J, Fromme P, Chapman HN, Pollack L, Tremblay L, Ourmazd A, Phillips GN, Schmidt M (2018). BMC Biol.

[100] Mehrabi P, Schulz EC, Agthe M, Horrell S, Bourenkov G, von Stetten D, Leimkohl JP, Schikora H, Schneider TR, Pearson AR, Tellkamp F, Miller RJD (2019). Nat Methods.

[101] Fuller FD, Gul S, Chatterjee R, Burgie ES, Young ID, Lebrette H, Srinivas V, Brewster AS, Michels-Clark T, Clinger JA, Andi B, Ibrahim M, Pastor E, de Lichtenberg C, Hussein R, Pollock CJ, Zhang M, Stan CA, Kroll T, Fransson T, Weninger C, Kubin M, Aller P, Lassalle L, Bräuer P, Miller MD, Amin M, Koroidov S, Roessler CG, Allaire M, Sierra RG, Docker PT, Glownia JM, Nelson S, Koglin JE, Zhu D, Chollet M, Song S, Lemke H, Liang M, Sokaras D, Alonso-Mori R, Zouni A, Messinger J, Bergmann U, Boal AK, Bollinger JM, Krebs C, Högbom M, Phillips GN, Vierstra RD, Sauter NK, Orville AM, Kern J, Yachandra VK, Yano J (2017). Nat Methods.

[102] Kwon H, Schrader TE, Ostermann A, Blakeley MP, Raven EL, Moody PCE (2020). Methods Enzymol.

[103] Habib MH, Rozeboom HJ, Fraaije MW (2019) Molecules 24 10.3390/molecules24071208 PMC6479361 30934796

[104] Shrestha R, Chen X, Ramyar KX, Hayati Z, Carlson EA, Bossmann SH, Song L, Geisbrecht BV, Li P (2016). ACS Catal.

[105] Dhankhar P, Dalal V, Mahto JK, Gurjar BR, Tomar S, Sharma AK, Kumar P (2020) Arch Biochem Biophys 693 10.1016/j.abb.2020.108590 32971035

[106] Rahmanpour R, Rea D, Jamshidi S, Fulop V, Bugg TD (2016). Arch Biochem Biophys.

[107] Zubieta C, Krishna SS, Kapoor M, Kozbial P, McMullan D, Axelrod HL, Miller MD, Abdubek P, Ambing E, Astakhova T, Carlton D, Chiu HJ, Clayton T, Deller MC, Duan L, Elsliger MA, Feuerhelm J, Grzechnik SK, Hale J, Hampton E, Han GW, Jaroszewski L, Jin KK, Klock HE, Knuth MW, Kumar A, Marciano D, Morse AT, Nigoghossian E, Okach L, Oommachen S, Reyes R, Rife CL, Schimmel P, van den Bedem H, Weekes D, White A, Xu Q, Hodgson KO, Wooley J, Deacon AM, Godzik A, Lesley SA, Wilson IA (2007). Proteins.

[108] Zubieta C, Joseph R, Krishna SS, McMullan D, Kapoor M, Axelrod HL, Miller MD, Abdubek P, Acosta C, Astakhova T, Carlton D, Chiu HJ, Clayton T, Deller MC, Duan L, Elias Y, Elsliger MA, Feuerhelm J, Grzechnik SK, Hale J, Han GW, Jaroszewski L, Jin KK, Klock HE, Knuth MW, Kozbial P, Kumar A, Marciano D, Morse AT, Murphy KD, Nigoghossian E, Okach L, Oommachen S, Reyes R, Rife CL, Schimmel P, Trout CV, van den Bedem H, Weekes D, White A, Xu Q, Hodgson KO, Wooley J, Deacon AM, Godzik A, Lesley SA, Wilson IA (2007). Proteins.

[109] Uchida T, Sasaki M, Tanaka Y, Ishimori K (2015). Biochemistry.

[110] Rai A, Klare JP, Reinke PYA, Englmaier F, Fohrer J, Fedorov R, Taft MH, Chizhov I, Curth U, Plettenburg O, Manstein DJ (2021) Int J Mol Sci 22 10.3390/ijms22126265 PMC8230527 34200865

[111] Brown ME, Barros T, Chang MC (2012). ACS Chem Biol.

[112] Yoshida T, Ogola HJ, Amano Y, Hisabori T, Ashida H, Sawa Y, Tsuge H, Sugano Y (2016). Proteins.

[113] Yoshida T, Tsuge H, Konno H, Hisabori T, Sugano Y (2011). FEBS J.

[114] Strittmatter E, Liers C, Ullrich R, Wachter S, Hofrichter M, Plattner DA, Piontek K (2013). J Biol Chem.

[115] Linde D, Pogni R, Canellas M, Lucas F, Guallar V, Baratto MC, Sinicropi A, Saez-Jimenez V, Coscolin C, Romero A, Medrano FJ, Ruiz-Duenas FJ, Martinez AT (2015). Biochem J.

[116] Strittmatter E, Serrer K, Liers C, Ullrich R, Hofrichter M, Piontek K, Schleicher E, Plattner DA (2015). Arch Biochem Biophys.

[117] Linde D, Canellas M, Coscolin C, Davo-Siguero I, Romero A, Lucas F, Ruiz-Duenas FJ, Guallar V, Martinez AT (2016). Catal Sci Technol.

[118] Fernandez-Fueyo E, Davo-Siguero I, Almendral D, Linde D, Baratto MC, Pogni R, Romero A, Guallar V, Martinez AT (2018). ACS Catal.

